# Early Maternal Prenatal Cannabis Use and Child Developmental Delays

**DOI:** 10.1001/jamanetworkopen.2024.40295

**Published:** 2024-10-18

**Authors:** Lyndsay A. Avalos, Nina Oberman, Stacey E. Alexeeff, Lisa A. Croen, Meghan N. Davignon, Sara R. Adams, Deborah Ansley, Christina D. Chambers, Kristin Steuerle, Kelly C. Young-Wolff

**Affiliations:** 1 Division of Research, Kaiser Permanente Northern California, Pleasanton; 2 Bernard J. Tyson Kaiser Permanente School of Medicine, Pasadena, California; 3 Pediatric Developmental Disabilities, Pediatric Subspecialties, Kaiser Permanente Roseville Medical Center, Roseville, California; 4 Regional Offices, Kaiser Permanente Northern California, Oakland; 5 Department of Pediatrics, School of Medicine, University of California, San Diego, La Jolla; 6 Kaiser Permanente Santa Rosa Medical Center, Santa Rosa, California; 7 Department of Psychiatry and Behavioral Sciences, University of California, San Francisco

## Abstract

**Importance:**

Maternal prenatal cannabis use is associated with adverse neonatal health effects, yet little is known about its association with child developmental outcomes.

**Objective:**

To evaluate associations between maternal prenatal cannabis use in early pregnancy and child early developmental delays.

**Design, Setting, and Participants:**

This cohort study included 119 976 children born to 106 240 unique individuals between January 2015 and December 2019 and followed up to aged 5.5 years or younger (through December 31, 2021) at Kaiser Permanente Northern California. Individuals were screened for prenatal cannabis use via self-report and urine toxicology at entrance into prenatal care (approximately 8- to 10-weeks’ gestation). Data were analyzed from February 2023 to March 2024.

**Exposure:**

Maternal prenatal cannabis use defined as any use (self-reported or by urine toxicology testing) and use frequency.

**Main Outcomes:**

Early developmental delays (speech and language disorders, motor delays, global delays) in children up to age 5.5 years defined by *International Statistical Classification of Diseases and Related Health Problems, Ninth Revision* and *Tenth Revision* diagnoses codes ascertained from electronic health records.

**Results:**

In this cohort of 119 976 pregnancies among 106 240 unique pregnant individuals, there were 29 543 Hispanic pregnancies (24.6%), 6567 non-Hispanic Black pregnancies (5.5%), 46 823 non-Hispanic White pregnancies (39.0%), 12 837 pregnancies (10.7%) to individuals aged 24 years or younger, and 10 365 pregnancies (8.6%) to individuals insured by Medicaid. Maternal prenatal cannabis use was documented for 6778 pregnancies (5.6%). Daily maternal prenatal cannabis use was reported for 618 pregnancies (0.5%), weekly for 722 pregnancies (0.6%), and monthly or less for 1617 pregnancies (1.3%). No association was observed between maternal prenatal cannabis use and child speech and language disorders (HR, 0.93; 95% CI, 0.84-1.03), global developmental delays (HR, 1.04; 95% CI, 0.68-1.59), or motor delays (HR, 0.86; 95% CI, 0.69-1.06). No association was detected between the frequency of maternal prenatal cannabis use and child early developmental delays.

**Conclusions and Relevance:**

In this cohort study, maternal prenatal cannabis use was not associated with an increased risk of child early developmental delays. Future research is needed to assess different patterns of cannabis use throughout pregnancy. Given the association between maternal prenatal cannabis use and other adverse outcomes, pregnant individuals should be educated on those risks.

## Key Points

**Question:**

Is maternal prenatal cannabis use during early pregnancy associated with child early developmental delays (ie, speech and language disorders, motor delays, global delays)?

**Findings:**

In this cohort study of 119 976 mother-child dyads, maternal cannabis use during early pregnancy was not associated with child early developmental delays in children aged 5.5 years or younger.

**Meaning:**

These findings suggest that maternal cannabis use in early pregnancy was not associated with an increased risk of child early developmental delays, but additional research on cannabis use throughout pregnancy, mode of administration, and product strength should be conducted.

## Teaser

This cohort study examines associations between maternal prenatal cannabis use in early pregnancy and early child developmental delays.

## Introduction

Cannabis use during pregnancy has been increasing in the US.^1,2^ A growing number of states across the US have legalized cannabis^3^ and studies have documented an increase in accessibility and acceptance of use.^4^ Thus, understanding how exposure during pregnancy affects child development is important.

A growing body of literature has documented associations between maternal prenatal cannabis use and adverse birth outcomes, including preterm birth and low birthweight.^5,6^ Additionally, cannabinoids can cross the placenta and enter the fetal bloodstream potentially disrupting fetal neurodevelopment. However, there has been little research assessing the association of maternal prenatal cannabis use and childhood developmental outcomes, specifically early developmental delays such as speech and language disorders, motor delays, and global delays.^7^ Studies evaluating speech and language outcomes report conflicting results with a study reporting worse verbal ability^8^ and others documenting no association with language and verbal ability.^8,9,10^ Similarly, inconsistent findings have been reported for motor skills. One study reported superior motor performance,^8^ another found worse processing speed and interhemispheric coordination on fine motor tasks,^11^ and others documented no association with motor ability or performance^9,11,12^ or psychomotor speed or eye-hand coordination.^13^ To our knowledge, global delays, such as delayed milestones, have not been evaluated in the context of maternal prenatal cannabis use. Furthermore, nearly all prior studies rely solely on self-reported cannabis use, which may underestimate the true prevalence of use during pregnancy and parent-reported developmental outcomes or the use of screeners rather than clinical diagnoses which may result in misclassification.^14^ This study evaluated the association between maternal cannabis use during early pregnancy and delays diagnosed in early childhood (speech and language, motor, and global) in a population universally screened for maternal prenatal cannabis use and early childhood developmental delays.

## Methods

### Setting

The study was set within Kaiser Permanente Northern California (KPNC), an integrated health care delivery system, which provides health care to approximately 4.6 million members with insurance coverage by Kaiser Foundation Health Plans, Medicaid, and/or Medicare Advantage. Clinical data are maintained within administrative and electronic health records (EHR).^15^ Pregnant patients are universally screened for prenatal substance use by both self-report (via a self-administered questionnaire) and a urine toxicology test to which they provide consent.^16^ As part of standard pediatrics care, children are screened for age-appropriate development at each well-baby or well-child visit. The institutional review board at KPNC approved the study and waived consent because data were deidentified. This cohort study followed the Strengthening the Reporting of Observational Studies in Epidemiology (STROBE) reporting guideline.

### Cohort

This retrospective birth cohort study included pregnant individuals with a singleton pregnancy and their children born between January 1, 2015, and December 31, 2019. Eligibility criteria included continuous KPNC health plan membership (ie, gaps of less than 3 months) from 1 year prior to pregnancy onset through delivery date, a minimum of 1 prenatal care visit, and a response to the cannabis use during pregnancy screening question and a prenatal toxicology test for tetrahydrocannabinol (THC) (eFigure 1 in Supplement 1). Pregnant individuals with a prenatal prescription fill for a teratogenic, antineoplastic, or antiepileptic drug (eAppendix 1 in Supplement 1), missing data on parity or address during pregnancy, and infants not enrolled in the health plan within 1 year of birth date or who died within 1 month of birth were excluded. Data for this study were obtained from KPNC’s administrative databases and EHR and California State Birth Certificates.

### Measures

#### Exposure

Maternal cannabis use during early pregnancy (primary exposure) was ascertained at entry into prenatal care (mean [SD] 8.2 (3.0) weeks’ gestation) and defined as maternal self-reported cannabis use since pregnancy and/or a positive urine toxicology test for cannabis. Frequency of prenatal cannabis use (secondary exposure) was ascertained from the self-report questionnaire and categorized as never, monthly or less, weekly, or daily. For individuals with a positive urine toxicology test for cannabis who self-reported never using cannabis since becoming pregnant, frequency of use was categorized as unknown frequency (eAppendix 1 in Supplement 1).

#### Outcomes

*International Statistical Classification of Diseases and Related Health Problems, Tenth Revision* (*ICD-10*) diagnosis codes and *Common Procedure Terminology* (*CPT*) codes were ascertained from the EHR to define 3 early developmental outcomes: speech and language disorders (*ICD-10*: F80.x, R47.X; *CPT*: 92507), motor delay (*ICD-10*: F82), and global developmental delay (*ICD-10*: F88, F89). Speech and language disorders were defined as at least 2 diagnoses on different dates by age 5.5 years, or 2 or more speech therapy sessions on different dates between age 1 year and 5.5 years. Motor delay and global developmental delays were defined as at least 2 diagnoses on 2 different dates by age 5.5 years.

#### Covariates

Maternal sociodemographic characteristics were ascertained from the EHR and birth certificates. Age at pregnancy onset (18 years or younger, 18 to 24 years, 25 to 30 years, 31 to 35 years, 36 years or older), parity (0, 1, more than 1, unknown), and insurance type (Medicaid vs other) were extracted from the EHR. The Neighborhood Deprivation Index (NDI),^17^ an indicator of neighborhood-level socioeconomic position, was calculated based on the earliest address in pregnancy. Self-reported race or ethnicity (Asian or Pacific Islander, Hispanic, non-Hispanic Black, non-Hispanic White, and other or unknown [including American Indian, Alaska Native, and multiracial individuals]) and self-reported educational attainment (high school or less, some college or technical school, college graduation, graduate school, unknown) were ascertained from the EHR and supplemented with birth certificate data when available. Race and ethnicity were included as a social construct due to known differences in the prevalence of cannabis use and incidence of child developmental delays by race and ethnicity.

Other noncannabis prenatal substance exposure included individual variables for alcohol, nicotine, opioids, stimulants, and anxiety or sleep medications, defined by maternal self-report of any use since pregnancy at entry to prenatal care, a positive toxicology test at entry to prenatal care, and/or pharmacy dispensation during pregnancy before the first prenatal visit date or prior to pregnancy with supply lasting through pregnancy onset (eAppendix 1 in Supplement 1). Adequacy of prenatal care was assessed using the Kotelchuck Month of Initiation Index and was categorized as inadequate (month 7 or more), intermediate (month 5 to 6), adequate (month 3 to 4), or adequate plus (month 1 to 2).^18^

Maternal comorbidities were defined by *ICD*-*9* and *ICD-10* codes (eAppendix 2 Supplement 1) and included diabetes (diagnosed in the 2 years prior to pregnancy onset); asthma, mood or anxiety disorders, other psychiatric disorders, chronic pain, and substance use disorders excluding cannabis-related disorders (diagnosed in the year prior to pregnancy onset through the first prenatal visit date); and nausea or vomiting during pregnancy (diagnosed from pregnancy onset through the first prenatal visit date). Antidepressant medication use was defined as a pharmacy dispensing during pregnancy before the first prenatal visit date or prior to pregnancy with supply lasting through pregnancy onset (eAppendix 1 in Supplement 1).

### Statistical Analysis

We fit Cox proportional hazards regression models to examine the association of maternal prenatal cannabis use with each developmental delay. Follow-up time began at birth, with age of the child (in months) as the time scale. Children were followed up until the outcome (first diagnosis or therapy session) or until censored at the end of health plan membership (ie, more than a 3-month gap in enrollment), the time of a missed well-child visit, death, end of the study period (December 31, 2021), or the maximum follow-up age of 66 months (5.5 years).

Attendance at well-child visits (part of standard pediatric care) was required for cohort follow-up to ensure equal opportunity for ascertainment of the outcome. Prior analyses identified differential attendance of well-child visits by maternal prenatal cannabis use.^19^ To account for the potential impact of informative censoring, we fit Cox models with inverse probability of censoring weights (IPCW).^20^ Time-varying stabilized weights were included for each time interval that the children were followed (eAppendix 3 in Supplement 1 for time intervals). To account for correlation of outcomes among children born to the same mother in separate pregnancies during the study, we used a marginal Cox model with a cluster term at the maternal level and robust standard errors. To determine the set of adjustment covariates, we developed a directed acyclic graph (DAG)^21^ with the DAGitty web application,^22^ using the best available evidence from the literature and subject matter expertise. The minimally sufficient set of variables identified by the DAG were included in the fully adjusted models (model 5) (eFigure 2 in Supplement 1).

We fit models sequentially to examine the degree of confounding by each set of covariates. All models adjusted for child age by using child age as the time scale of the Cox model. Model 1 did not include any confounders. Model 2 was adjusted for maternal sociodemographic characteristics. Model 3 was additionally adjusted for maternal noncannabis prenatal substance use. Model 4 was additionally adjusted for month of prenatal care initiation. Model 5 (fully adjusted) was additionally adjusted for maternal comorbidities. Analyses were conducted from February 2023 to March 2024 using R version 4.0.2 (R Project for Statistical Computing). Tests were 2-sided, and *P* < .05 was considered statistically significant.

We conducted 3 additional analyses to assess the sensitivity of our exposure. The sensitivity of our exposure was assessed by defining prenatal cannabis as determined only by self-reported use, and then only by urine toxicology results. The third analysis excluded pregnancies with non-cannabis prenatal substance use. To assess the potential effects of selection bias, we conducted a fourth sensitivity analysis including all pregnancies with any enrollment at KPNC during the pregnancy period (eFigure 5 in Supplement 1).

## Results

The cohort included 119 976 pregnancies (among 106 240 unique pregnant individuals). The sample included 32 793 Asian or Pacific Islander pregnancies (27.3%), 29 543 Hispanic pregnancies (24.6%), 6567 non-Hispanic Black pregnancies (5.5%), and 46 823 non-Hispanic White pregnancies (39.0%); 12 837 pregnancies (10.7%) were to individuals aged 24 years or less; 25 568 (21.3%) had at least 2 previous live births, and 10 365 (8.6%) were insured by Medicaid (Table 1). Maternal prenatal cannabis use was identified (via self-report or urine toxicology) for 6778 (5.6%) pregnancies. Of those, 1785 (26.3%) were positive by both self-report and toxicology results, 3821 (56.4%) by toxicology test only, and 1172 (17.3%) by self-report only. A total of 618 pregnancies (0.5%) identified with maternal prenatal cannabis use were to individuals who reported daily use, 722 (0.6%) who reported weekly use, and 1617 (1.3%) who reported monthly or less use. Urine toxicology tests were conducted at mean (SD) of 9.4 (3.6) weeks’ gestation; 5606 pregnant individuals (4.7%) tested positive for THC. Overall, 115 363 pregnancies (96.2%) were screened in the first trimester. Maternal sociodemographic and clinical characteristics by prenatal cannabis use are shown in Table 1 and eTable 1 in Supplement 1.

**Table 1.** Pregnancy Characteristics by Prenatal Cannabis Use Among Live Births at Kaiser Permanente Northern California, 2015-2019

Characteristics	Pregnancies, No. (%)		
	Total^a^	Prenatal cannabis use	
		No	Yes
Total, No.	119 976	113 198	6778
Maternal sociodemographic characteristics			
Race and ethnicity			
Asian and Pacific Islander	32 793 (27.3)	32 334 (28.6)	459 (6.8)
Hispanic	29 543 (24.6)	27 600 (24.4)	1943 (28.7)
Non-Hispanic Black	6567 (5.5)	5218 (4.6)	1349 (19.9)
Non-Hispanic White	46 823 (39.0)	44 209 (39.1)	2614 (38.6)
Other or unknown^b^	4250 (3.5)	3837 (3.4)	413 (6.1)
Age at pregnancy onset, y			
<18	597 (0.5)	466 (0.4)	131 (1.9)
18-24	12 240 (10.2)	10 046 (8.9)	2194 (32.4)
25-30	38 621 (32.2)	36 404 (32.2)	2217 (32.7)
31-35	45 993 (38.3)	44 440 (39.3)	1553 (22.9)
≥36	22 525 (18.8)	21 842 (19.3)	683 (10.1)
Parity			
0	49 794 (41.5)	46 123 (40.7)	3671 (54.2)
1	44 614 (37.2)	42 710 (37.7)	1904 (28.1)
≥2	25 568 (21.3)	24 365 (21.5)	1203 (17.7)
Insurance type			
Medicaid	10 365 (8.6)	8641 (7.6)	1724 (25.4)
Non-Medicaid	109 611 (91.4)	104 557 (92.4)	5054 (74.6)
Education level			
High school or less	15 842 (13.2)	13 869 (12.3)	1973 (29.1)
Some college	34 731 (28.9)	31 644 (28.0)	3087 (45.5)
College graduate	40 408 (33.7)	39 303 (34.7)	1105 (16.3)
Graduate school	26 291 (21.9)	25 845 (22.8)	446 (6.6)
Unknown	2704 (2.3)	2537 (2.2)	167 (2.5)
Neighborhood Deprivation Index			
Q1, least deprived	29 988 (25.0)	29 096 (25.7)	892 (13.2)
Q2	30 000 (25.0)	28 662 (25.3)	1338 (19.7)
Q3	30 002 (25.0)	28 174 (24.9)	1828 (27.0)
Q4, most deprived	29 986 (25.0)	27 266 (24.1)	2720 (40.1)
Other (noncannabis) prenatal substance use^c^			
Alcohol use	11 107 (9.3)	9741 (8.6)	1366 (20.2)
Nicotine use	4127 (3.4)	2825 (2.5)	1302 (19.2)
Anxiety or sleep medication use	3336 (2.8)	2853 (2.5)	483 (7.1)
Stimulant use	874 (0.7)	659 (0.6)	215 (3.2)
Opioid use	2474 (2.1)	2100 (1.9)	374 (5.5)
Prenatal care utilization			
Kotelchuck Month of Initiation Index			
Inadequate (month ≥7)	435 (0.4)	371 (0.3)	64 (0.9)
Intermediate (month 5-6)	1209 (1.0)	1073 (0.9)	136 (2.0)
Adequate (month 3-4)	32 032 (26.7)	30 140 (26.6)	1892 (27.9)
Adequate plus (month 1-2)	86 300 (71.9)	81 614 (72.1)	4686 (69.1)
Maternal comorbidities			
Diabetes (type I or II)^d^	1728 (1.4)	1642 (1.5)	86 (1.3)
Asthma^e^	13 093 (10.9)	11 832 (10.5)	1261 (18.6)
Nausea or vomiting in pregnancy^f^	13 200 (11.0)	11 586 (10.2)	1614 (23.8)
Mood or anxiety disorders^e^	15 792 (13.2)	14 018 (12.4)	1774 (26.2)
Other psychiatric disorders^e^	3268 (2.7)	2782 (2.5)	486 (7.2)
Antidepressant use in pregnancy^g^	5608 (4.7)	4978 (4.4)	630 (9.3)
Chronic pain^e^	4524 (3.8)	4038 (3.6)	486 (7.2)
Substance use disorders^e,h^	3769 (3.1)	2709 (2.4)	1060 (15.6)
Infant characteristics			
Infant sex			
Male	61 619 (51.4)	58 191 (51.4)	3428 (50.6)
Female	58 357 (48.6)	55 007 (48.6)	3350 (49.4)
Secondary exposure			
Frequency of prenatal cannabis use			
None	113 198 (94.4)	113 198 (100.0)	0
Monthly or less	1617 (1.3)	0	1617 (23.9)
Weekly	722 (0.6)	0	722 (10.7)
Daily	618 (0.5)	0	618 (9.1)
Unknown frequency^i^	3821 (3.2)	0	3821 (56.4)

^a^ The cohort consisted of 119 976 unique pregnancies in 106 240 unique pregnant individuals.

^b^ Other race or ethnicity includes American Indian, Alaska Native, and multiracial individuals.

^c^ Assessed at entrance to prenatal care or prescription filled during pregnancy through date of first prenatal visit. See eAppendix 2 in Supplement 1 for full details.

^d^ In 2 years prior to pregnancy onset.

^e^ In year prior to pregnancy onset or during pregnancy through date of first prenatal visit.

^f^ Through date of first prenatal visit.

^g^ Prescription fill before pregnancy with supply lasting through pregnancy onset or during pregnancy through date of first prenatal visit.

^h^ Excludes cannabis-related disorders.

^i^ Positive urine toxicology test for tetrahydrocannabinol, but reported no cannabis use since becoming pregnant.

The median (IQR) age of follow-up was 39 (26-46) months (39 [26-47] months for children not exposed to maternal prenatal cannabis use and 29 [15-42] months for those exposed). The cumulative incidence of speech and language disorders among children up to age 5.5 years was 11.1% (95% CI, 10.9%-11.4%), with a first diagnosis or therapy session at mean (SD) age of 25 (9) months. The cumulative incidence of global developmental delay was 0.5% (95% CI, 0.5%-0.6%) among children up to age 5.5 years, with a first diagnosis at mean (SD) age of 23 (11) months. The cumulative incidence of motor delay up to age 5.5 years was 2.2% (95% CI, 2.1%-2.3%), with a first diagnosis at mean (SD) age of 13 (11) months. Cumulative incidences by prenatal cannabis use are presented in Table 2.

**Table 2.** Hazard Ratios for Associations Between Prenatal Cannabis Use and Early Developmental Delays (n = 119 976)

Outcome	Cumulative incidence (95% CI), %	HR (95% CI)				
		Model 1^a^	Model 2^b^	Model 3^c^	Model 4^d^	Model 5^e^
Speech and language disorders	11.1 (10.9-11.4)	NA	NA	NA	NA	NA
Prenatal cannabis use						
No	11.1 (10.8-11.4)	1 [Reference]	1 [Reference]	1 [Reference]	1 [Reference]	1 [Reference]
Yes	12.2 (10.8-13.7)	1.01 (0.91-1.10)	0.96 (0.86-1.06)	0.95 (0.85-1.06)	0.95 (0.85-1.06)	0.93 (0.84-1.03)
Global delay	0.5 (0.5-0.6)	NA	NA	NA	NA	NA
Prenatal cannabis use						
No	0.5 (0.5-0.6)	1 [Reference]	1 [Reference]	1 [Reference]	1 [Reference]	1 [Reference]
Yes	0.8 (0.5-1.2)	1.38 (0.94-2.03)	1.08 (0.71-1.64)	1.07 (0.70-1.65)	1.07 (0.69-1.64)	1.04 (0.68-1.59)
Motor delay	2.2 (2.1-2.3)	NA	NA	NA	NA	NA
Prenatal cannabis use						
No	2.2 (2.1-2.3)	1 [Reference]	1 [Reference]	1 [Reference]	1 [Reference]	1 [Reference]
Yes	2.1 (1.5-2.7)	0.84 (0.69-1.00)	0.91 (0.74-1.13)	0.89 (0.72-1.11)	0.89 (0.72-1.11)	0.86 (0.69-1.06)

Abbreviations: HR, hazard ratio; NA, not applicable.

^a^ Unadjusted Cox proportional hazards model with inverse-probability of censoring weights and maternal-level cluster term.

^b^ Adjusted for sociodemographic characteristics (age at pregnancy onset, race or ethnicity, education, Neighborhood Deprivation Index, parity, Medicaid status).

^c^ Additionally adjusted for other noncannabis prenatal substance use (alcohol, nicotine, opioids, anxiety or sleep medication, and stimulants).

^d^ Additionally adjusted for month of prenatal care initiation.

^e^ Additionally adjusted for maternal medical and mental health comorbidities (asthma, diabetes, nausea or vomiting during pregnancy, mood or anxiety disorders, other psychiatric disorders, substance use disorders, antidepressant use, chronic pain).

Maternal prenatal cannabis use was not associated with speech and language disorders in model 1 (hazard ratio [HR]: 1.01; 95% CI, 0.91-1.10) (Table 2). Results were similar as covariates were added in models 2 to 5 (model 5: HR, 0.93, 95% CI, 0.84-1.03). Self-reported frequency of maternal prenatal cannabis use was not associated with speech and language disorders (Figure). The sensitivity analysis defining prenatal cannabis use with only self-report data produced results similar to the main analysis (eTable 2 in Supplement 1). The sensitivity analysis defining prenatal cannabis use with only urine toxicology test data produced a modest inverse association with speech and language disorders (HR, 0.88; 95% CI, 0.78-0.99) (eTable 3 in Supplement 1). The sensitivity analysis restricted to pregnancies with no noncannabis substance use produced an inverse association with self-reported monthly or less cannabis use and speech and language disorders (HR, 0.68; 95% CI, 0.46-1.00) (eFigure 3 in Supplement 1).

**Figure.**
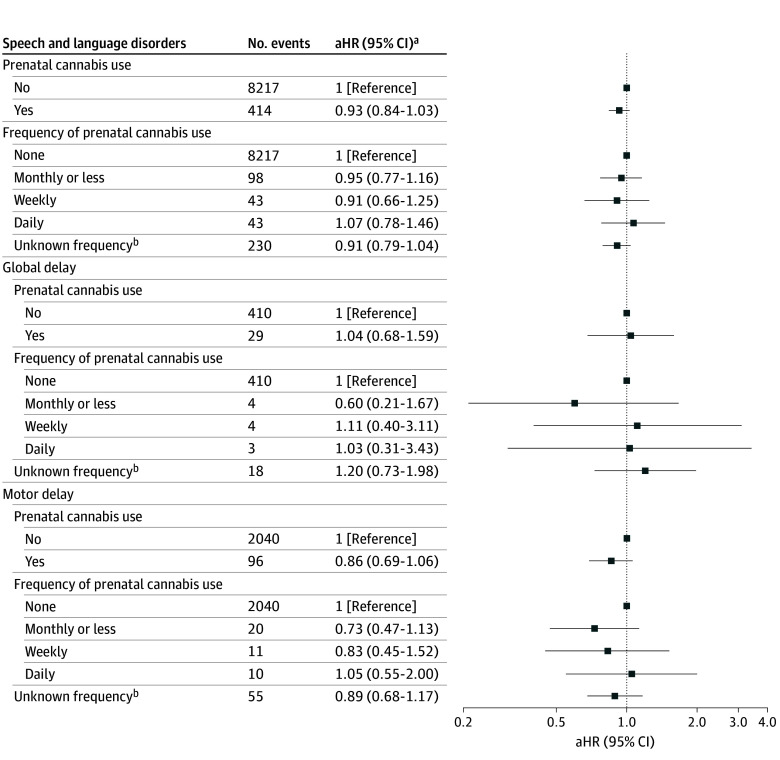
Hazard Ratios for Associations Between Prenatal Cannabis Use and Early Developmental Delays, Overall and by Frequency of Prenatal Cannabis Use aHR indicates adjusted hazard ratio. ^a^Adjusted for sociodemographic variables, other noncannabis substance use, prenatal care initiation, and medical and mental health comorbidities ^b^Positive urine toxicology test for tetrahydrocannabinol but reported no cannabis use since becoming pregnant.

Although a suggestive increased risk of global developmental delay was found with any prenatal cannabis use in model 1 (HR, 1.38; 95% CI, 0.94-2.03), after adjusting for maternal demographic characteristics the association was attenuated and remained not statistically significant (model 2: HR, 1.08; 95% CI, 0.71-1.64). Adjustment for all potential confounders also demonstrated no statistically significant association (model 5: HR, 1.04; 95% CI, 0.68-1.59) (Table 2). There was no association between self-reported frequency of cannabis use and global developmental delay (Figure). Similarly, no significant associations were observed for global developmental delay in sensitivity analyses defining prenatal cannabis use by self-report data only and by urine toxicology test data only (eTables 2 and 3 in Supplement 1) and in the sensitivity analysis restricting to pregnancies with no noncannabis substance use (eFigure 3 in Supplement 1).

Although a suggestive decreased risk of motor delay was found with any maternal prenatal cannabis use in model 1 (HR, 0.84; 95% CI, 0.69-1.00), after adjustment for all potential confounders the association was not statistically significant (model 5: HR, 0.86; 95% CI, 0.69-1.06) (Table 2). There was no association between self-reported frequency of cannabis use and motor delay (Figure). No statistically significant associations were observed for motor delay in the sensitivity analyses (eTables 2 and 3 and eFigure 3 in Supplement 1). The sensitivity analysis with no continuous enrollment requirement in the year prior to or during pregnancy produced results generally similar to the main analyses (eFigures 4 and 5 in Supplement 1).

## Discussion

In this large, population-based longitudinal birth cohort study, maternal cannabis use during early pregnancy was not associated with speech and language disorders, global delay, or motor delay. These associations were relatively stable across multiple sets of adjustment for potential confounders. Sensitivity analyses also largely support findings from the main analyses, except for a suggested modest inverse association with speech and language disorders when defining cannabis use based only on the urine toxicology results. Understanding the associations between maternal prenatal cannabis use and these early developmental outcomes is important because they may be early indicators of other salient developmental outcomes including autism spectrum disorder (ASD), attention-deficit/hyperactivity disorder (ADHD), and intellectual disability.^23,24,25^

Our findings are consistent with the majority of previous studies evaluating maternal prenatal cannabis use and various characteristics of speech and language development.^8,9,10^ The previous research evaluating speech and language outcomes was based on data from the Ottawa Prenatal Prospective Study (OPPS) which ascertained prenatal cannabis use from maternal self-report. The study population was recruited in 1978 representing a time of lower potency cannabis products and the largest analytic sample evaluating speech and language disorders included 272 parent-child dyads.^9^ Maternal prenatal cannabis use was not associated with several speech and language outcomes (eg, verbal ability, language ability) at age 12 and 24 months,^10^ and 3,^8^ 4,^8^ or 5 to 6 years.^9^ Contrary to our findings, an association between heavy cannabis use and poor verbal ability at 4 years of age was documented in an analysis.^8^

An association between maternal prenatal cannabis use and superior motor performance in children aged 3 years was documented in an OPPS analysis,^8^ in contrast with the majority of the research evaluating motor developmental outcomes, including ours, which did not find an association. Four analyses conducted on data from the OPPS and the Maternal Health Practices and Child Development (MHPCD) studies found no association between maternal prenatal cannabis use and various motor skill traits including fine motor skills,^11^ psychomotor speed and eye-hand coordination,^13^ and motor ability.^8,12^ In contrast, an analysis from the MHPCD study reported worse interhemispheric coordination on fine motor tasks. This study evaluated outcomes at age 16 years, suggesting poorer motor outcomes may not arise until adolescence.^11^

Future research is needed to understand the inverse association with speech and language disorders that emerged with a positive urine toxicology test. Our dose-response analyses of self-report data and speech and language disorders showed null associations, and the estimate for self-reported daily use was in the positive direction, contrasting the urine toxicology findings. Because our routine toxicology screening only documents yes or no use rather than concentration of THC, we were unable to investigate the dose-response association of toxicology results. Our prior study comparing self-report and toxicology results found that toxicology screening detected 65.8% of women who self-reported cannabis use, with greater detection by toxicology for self-reported daily (83.9%) and weekly (77.4%) than monthly or less use (54.1%).^14^ Future studies with more detailed data on frequency and duration of use and concentration of THC may be able to determine whether there is any association or whether the association is null.

Maternal prenatal cannabis use has been associated with adverse neonatal outcomes^26,27,28^ and increased child psychopathology.^29,30^ Furthermore, the underlying neurobiology and animal models suggest potential health risks. Animal models indicate that THC exposure may disrupt normal brain development and function by interfering with the endocannabinoid system^31^ resulting in long-lasting neurodevelopmental and behavioral abnormalities.^32,33,34,35^ Epidemiologic research has documented changes to brain structure and function with prenatal cannabis exposure.^36,37,38^ Despite our findings of no increased risks for early developmental delays, past evidence for adverse fetal and neonatal outcomes indicate that American College of Obstetrics and Gynecology and American Academy of Pediatrics recommendations for pregnant individuals and those considering pregnancy to discontinue cannabis use should be followed.^39^

We note several strengths. This study included the largest number of pregnancies exposed to maternal cannabis use, and 1 of the largest cohorts to evaluate the association between maternal prenatal cannabis use and early developmental delays. The population of pregnant individuals was universally screened for cannabis use at entrance to prenatal care via urine toxicology and self-report, and children were routinely screened at multiple time points for developmental delays reducing recall bias and misclassification. Our study used diagnosis codes prospectively recorded in electronic medical records to identify early developmental delays. Diagnoses codes represent a more rigorous assessment of delay than used in previous research, which has relied on developmental screeners and parental report which are less accurate and informative for clinical care. To our knowledge, this analysis is among the first to assess infant global delay in relation to maternal prenatal cannabis use. We used rigorous methods through the development of a DAG to reduce potential bias and residual confounding and included many potential confounders captured through EHR data. We conducted sensitivity analyses to address variance in exposure classification by self-report and urine toxicology and confounding by cooccurrence of other maternal prenatal substance use which all produced similar results. Finally, we highlight the study sample was racially, ethnically, and geographically diverse.

### Limitations

This study has limitations. Maternal prenatal cannabis use was measured at a single time point (ie, entry into prenatal care). While research indicates the prevalence of cannabis use decreases by nearly half over the course of pregnancy (5.3% in the first trimester to 2.5% in the second and third trimester),^2^ we highlight this is an avenue for future research. Similarly, evaluation of exposure in the first trimester is relevant given it is widely recognized as a critical period for brain development and may reflect an important period for early developmental delays.^40^ We also did not have information on cannabis potency, mode of cannabis consumption (eg, smoking, vaping), or the concentration of THC detected in the positive toxicology screens. Children of individuals who reported cannabis use during pregnancy are less likely to attend well-child visits in early childhood^41^ and more likely to end their insurance coverage with KPNC, reducing their opportunity to be diagnosed with an early developmental delay. While we used rigorous statistical techniques, including censoring and applying IPCW, it may not have adequately addressed differential follow-up. If individuals who used cannabis during pregnancy are less likely to identify and/or report delays leading to an underdiagnosis of early developmental delays in their children, it could result in residual confounding and biased estimates. Finally, California legalized cannabis for adult use in 2016 and findings may not be generalizable to patients or outside of the state. Yet, we highlight the US population prevalence of maternal prenatal cannabis use (3% to 7%)^2,19^ is similar to the prevalence documented in the current study.

## Conclusions

This study did not find evidence of an association between maternal cannabis use in early pregnancy and child developmental delays up to age 5.5 years. Additional studies are needed to evaluate cannabis use throughout pregnancy, mode of administration and product strength, as well as potential factors that may mitigate adverse associations and neurodevelopmental outcomes that may emerge later in childhood. Given the association with other adverse neonatal outcomes and documented changes to brain structure and function, pregnant individuals and those considering pregnancy should be educated on the risks of prenatal cannabis use.

## Notes

Supplement 1. **eAppendix 1.** Ascertainment of Maternal Prenatal Cannabis Use and Other Substance Use **eAppendix 2.** Ascertainment of Maternal Medical and Mental Health Comorbidities **eAppendix 3.** Well-Child Visits and Censoring Intervals **eTable 1.** Pregnancy Characteristics Stratified by Reported Frequency of Prenatal Cannabis Use **eTable 2.** Hazard Ratios for Associations Between Prenatal Cannabis Use and Early Developmental Delays, Measured by Self-Report vs Urine Toxicology Results **eTable 3.** Hazard Ratios for Associations Between Prenatal Cannabis Use Measured by Urine Toxicology Test and Early Developmental Delays (n=119 976) **eFigure 1.** Flow Diagram of Study Inclusion for Infants Born to Pregnant Individuals Screened for Prenatal Substance Use at Kaiser Permanente Northern California (KPNC) From 2015 to 2019 **eFigure 2.** Directed Acyclic Graph (DAG) Depicting Relationships Between Prenatal Cannabis Use, Confounders, and Early Developmental Delays **eFigure 3.** Hazard Ratios for Associations Between Prenatal Cannabis Use and Early Developmental Delays, Overall and by Frequency of Use, Among Pregnancies With No Other Prenatal Substance Use **eFigure 4.** Hazard Ratios for Associations Between Prenatal Cannabis Use and Early Developmental Delays, Overall and by Frequency of Use, Among Cohort With No Continuous Enrollment Requirement and Either Prenatal Substance Use Screening Questionnaire or Prenatal Urine Toxicology Test Completed **eFigure 5.** Cohort Assembly of Infants Born to Pregnant Individuals Screened for Prenatal Substance Use at Kaiser Permanente Northern California (KPNC) from 2015 to 2019, With No Continuous Enrollment Requirement and Either Prenatal Substance Use Screening Questionnaire or Prenatal Urine Toxicology Test Completed

Supplement 2.
Data Sharing Statement

## References

[1] Young-Wolff KC, Tucker LY, Alexeeff S, . Trends in self-reported and biochemically tested marijuana use among pregnant females in California from 2009-2016. JAMA. 2017;318(24):2490-2491. doi:10.1001/jama.2017.17225 29279917 PMC5769923

[2] Volkow ND, Han B, Compton WM, McCance-Katz EF. Self-reported medical and nonmedical cannabis use among pregnant women in the United States. JAMA. 2019;322(2):167-169. doi:10.1001/jama.2019.7982 31211824 PMC6582258

[3] Hanson K, Garcia A. State medical cannabis laws. National Conference of State Legislatures. Accessed September 12, 2024. https://www.ncsl.org/health/state-medical-cannabis-laws

[4] Young-Wolff KC, Foti TR, Green A, . Perceptions about cannabis following legalization among pregnant individuals with prenatal cannabis use in California. JAMA Netw Open. 2022;5(12):e2246912. doi:10.1001/jamanetworkopen.2022.46912 36515947 PMC9856570

[5] Avalos LA, Adams SR, Alexeeff SE, . Neonatal outcomes associated with in utero cannabis exposure: a population-based retrospective cohort study. Am J Obstet Gynecol. 2023;231(1)132.e1-132.e13. doi:10.1016/j.ajog.2023.11.1232 38029850 PMC11128475

[6] Metz TD, Allshouse AA, McMillin GA, . Cannabis exposure and adverse pregnancy outcomes related to placental function. JAMA. 2023;330(22):2191-2199. doi:10.1001/jama.2023.21146 38085313 PMC10716715

[7] Sujan AC, Young-Wolff KC, Avalos LA. In-utero cannabis exposure and long-term psychiatric and neurodevelopmental outcomes: the limitations of existing literature and recommendations for future research. Birth Defects Res. 2022;114(13):689-713. doi:10.1002/bdr2.2060 35708102 PMC9357094

[8] Fried PA, Watkinson B. 36- and 48-Month neurobehavioral follow-up of children prenatally exposed to marijuana, cigarettes, and alcohol. J Dev Behav Pediatr. 1990;11(2):49-58. doi:10.1097/00004703-199004000-00003 2324288

[9] Fried PA, Watkinson B, Gray R. A follow-up study of attentional behavior in 6-year-old children exposed prenatally to marihuana, cigarettes, and alcohol. Neurotoxicol Teratol. 1992;14(5):299-311. doi:10.1016/0892-0362(92)90036-A 1454038

[10] Fried PA, Watkinson B. 12- and 24-Month neurobehavioural follow-up of children prenatally exposed to marihuana, cigarettes and alcohol. Neurotoxicol Teratol. 1988;10(4):305-313. doi:10.1016/0892-0362(88)90032-3 3226373

[11] Willford JA, Chandler LS, Goldschmidt L, Day NL. Effects of prenatal tobacco, alcohol and marijuana exposure on processing speed, visual-motor coordination, and interhemispheric transfer. Neurotoxicol Teratol. 2010;32(6):580-588. doi:10.1016/j.ntt.2010.06.004 20600845 PMC2975798

[12] Fried PA, O’Connell CM, Watkinson B. 60- and 72-Month follow-up of children prenatally exposed to marijuana, cigarettes, and alcohol: cognitive and language assessment. J Dev Behav Pediatr. 1992;13(6):383-391. doi:10.1097/00004703-199212000-00001 1469105

[13] Richardson GA, Ryan C, Willford J, Day NL, Goldschmidt L. Prenatal alcohol and marijuana exposure: effects on neuropsychological outcomes at 10 years. Neurotoxicol Teratol. 2002;24(3):309-320. doi:10.1016/S0892-0362(02)00193-9 12009486

[14] Young-Wolff KC, Sarovar V, Tucker LY, . Validity of self-reported cannabis use among pregnant females in northern California. J Addict Med. 2020;14(4):287-292. doi:10.1097/ADM.0000000000000581 31688149 PMC7931632

[15] Escobar GJ, Plimier C, Greene JD, Liu V, Kipnis P. Multiyear rehospitalization rates and hospital outcomes in an integrated health care system. JAMA Netw Open. 2019;2(12):e1916769-e1916769. doi:10.1001/jamanetworkopen.2019.16769 31800072 PMC6902762

[16] Taillac C, Goler N, Armstrong MA, Haley K, Osejo V. Early start: an integrated model of substance abuse intervention for pregnant women. Perm J. 2007;11(3):5-11. doi:10.7812/TPP/07-013 21461106 PMC3057720

[17] Messer LC, Laraia BA, Kaufman JS, . The development of a standardized neighborhood deprivation index. J Urban Health. 2006;83(6):1041-1062. doi:10.1007/s11524-006-9094-x 17031568 PMC3261293

[18] Kotelchuck M. An evaluation of the Kessner Adequacy of Prenatal Care Index and a proposed Adequacy of Prenatal Care Utilization Index. Am J Public Health. 1994;84(9):1414-1420. doi:10.2105/AJPH.84.9.1414 8092364 PMC1615177

[19] Avalos LA, Oberman N, Alexeeff SE, . Association between maternal prenatal cannabis use and missed child preventive care visits in an integrated health care delivery system in Northern California. Prev Med. 2023;175:107716. doi:10.1016/j.ypmed.2023.107716 37775081 PMC10849893

[20] Howe CJ, Cole SR, Lau B, Napravnik S, Eron JJ Jr. Selection bias due to loss to follow up in cohort studies. Epidemiology. 2016;27(1):91-97. doi:10.1097/EDE.0000000000000409 26484424 PMC5008911

[21] VanderWeele TJ, Hernán MA, Robins JM. Causal directed acyclic graphs and the direction of unmeasured confounding bias. Epidemiology. 2008;19(5):720-728. doi:10.1097/EDE.0b013e3181810e29 18633331 PMC4242711

[22] Textor J, van der Zander B, Gilthorpe MS, Liśkiewicz M, Ellison GT. Robust causal inference using directed acyclic graphs: the R package ‘dagitty’. Int J Epidemiol. 2016;45(6):1887-1894. doi:10.1093/ije/dyw341 28089956

[23] LeBarton ES, Landa RJ. Infant motor skill predicts later expressive language and autism spectrum disorder diagnosis. Infant Behav Dev. 2019;54:37-47. doi:10.1016/j.infbeh.2018.11.003 30557704

[24] McFayden TC, Rutsohn J, Cetin G, ; IBIS Network. White matter development and language abilities during infancy in autism spectrum disorder. Mol Psychiatry. 2024. doi:10.1038/s41380-024-02470-3 38383768 PMC11336031

[25] Havdahl A, Farmer C, Surén P, . Attainment and loss of early social-communication skills across neurodevelopmental conditions in the Norwegian Mother, Father and Child Cohort Study. J Child Psychol Psychiatry. 2024;65(5):610-619. doi:10.1111/jcpp.13792 36973172 PMC10522798

[26] Marchand G, Masoud AT, Govindan M, . Birth outcomes of neonates exposed to marijuana in utero: a systematic review and meta-analysis. JAMA Netw Open. 2022;5(1):e2145653. doi:10.1001/jamanetworkopen.2021.45653 35084479 PMC8796018

[27] Baía I, Domingues RMSM. The effects of cannabis use during pregnancy on low birth weight and preterm birth: a systematic review and meta-analysis. Am J Perinatol. 2024;41(1):17-30. doi:10.1055/a-1911-3326 35901851

[28] Lo JO, Shaw B, Robalino S, . Cannabis use in pregnancy and neonatal outcomes: a systematic review and meta-analysis. Cannabis Cannabinoid Res. 2024;9(2):470-485. doi:10.1089/can.2022.0262 36730710 PMC11262585

[29] Day NL, Goldschmidt L, Day R, Larkby C, Richardson GA. Prenatal marijuana exposure, age of marijuana initiation, and the development of psychotic symptoms in young adults. Psychol Med. 2015;45(8):1779-1787. doi:10.1017/S0033291714002906 25534593 PMC8128137

[30] Paul SE, Hatoum AS, Fine JD, . Associations between prenatal cannabis exposure and childhood outcomes: results from the ABCD study. JAMA Psychiatry. 2021;78(1):64-76. doi:10.1001/jamapsychiatry.2020.2902 32965490 PMC7512132

[31] Biegon A, Kerman IA. Autoradiographic study of pre- and postnatal distribution of cannabinoid receptors in human brain. Neuroimage. 2001;14(6):1463-1468. doi:10.1006/nimg.2001.0939 11707102

[32] Campolongo P, Trezza V, Ratano P, Palmery M, Cuomo V. Developmental consequences of perinatal cannabis exposure: behavioral and neuroendocrine effects in adult rodents. Psychopharmacology (Berl). 2011;214(1):5-15. doi:10.1007/s00213-010-1892-x 20556598 PMC3045519

[33] Campolongo P, Trezza V, Cassano T, . Perinatal exposure to delta-9-tetrahydrocannabinol causes enduring cognitive deficits associated with alteration of cortical gene expression and neurotransmission in rats. Addict Biol. 2007;12(3-4):485-495. doi:10.1111/j.1369-1600.2007.00074.x 17578508

[34] Trezza V, Vanderschuren LJ. Bidirectional cannabinoid modulation of social behavior in adolescent rats. Psychopharmacology (Berl). 2008;197(2):217-227. doi:10.1007/s00213-007-1025-3 18058088

[35] Brake SC, Hutchings DE, Morgan B, Lasalle E, Shi T. Delta-9-tetrahydrocannabinol during pregnancy in the rat: II. Effects on ontogeny of locomotor activity and nipple attachment in the offspring. Neurotoxicol Teratol. 1987;9(1):45-49. doi:10.1016/0892-0362(87)90069-9 3041193

[36] El Marroun H, Tiemeier H, Franken IH, . Prenatal cannabis and tobacco exposure in relation to brain morphology: a prospective neuroimaging study in young children. Biol Psychiatry. 2016;79(12):971-979. doi:10.1016/j.biopsych.2015.08.024 26422004

[37] Smith AM, Mioduszewski O, Hatchard T, Byron-Alhassan A, Fall C, Fried PA. Prenatal marijuana exposure impacts executive functioning into young adulthood: an fMRI study. Neurotoxicol Teratol. 2016;58:53-59. doi:10.1016/j.ntt.2016.05.010 27263090

[38] Smith AM, Fried PA, Hogan MJ, Cameron I. Effects of prenatal marijuana on visuospatial working memory: an fMRI study in young adults. Neurotoxicol Teratol. 2006;28(2):286-295. doi:10.1016/j.ntt.2005.12.008 16473495

[39] American College of Obstetricians and Gynecologists Committee on Obstetric Practice. Committee opinion No. 637: marijuana use during pregnancy and lactation. Obstet Gynecol. 2015;126(1):234-238. doi:10.1097/01.AOG.0000467192.89321.a6 26241291

[40] Organization of Teratology Information Specialists. Critical Periods of Development. Mother to Baby. https://www.ncbi.nlm.nih.gov/books/NBK582659/35951922

[41] Young-Wolff KC, Sarovar V, Tucker LY, . Self-reported daily, weekly, and monthly cannabis use among women before and during pregnancy. JAMA Netw Open. 2019;2(7):e196471. doi:10.1001/jamanetworkopen.2019.6471 31322686 PMC6646980

