# Supplementary material for: Early Maternal Prenatal Cannabis Use and Child Developmental Delays
Source: JAMA Netw Open. 2024 Oct 18;7(10):e2440295. doi: 10.1001/jamanetworkopen.2024.40295 (PMC11581621; doi:10.1001/jamanetworkopen.2024.40295)
Supplement: Supplement 1. — eAppendix 1. Ascertainment of Maternal Prenatal Cannabis Use and Other Substance Use eAppendix 2. Ascertainment of Maternal Medical and Mental Health Comorbidities eAppendix 3. Well-Child Visits and Censoring Intervals eTable 1. Pregnancy Characteristics Stratified by Reported Frequency of Prenatal Cannabis Use eTable 2. Hazard Ratios for Associations Between Prenatal Cannabis Use and Early Developmental Delays, Measured by Self-Report vs Urine Toxicology Results eTable 3. Hazard Ratios for Associations Between Prenatal Cannabis Use Measured by Urine Toxicology Test and Early Developmental Delays (n=119 976) eFigure 1. Flow Diagram of Study Inclusion for Infants Born to Pregnant Individuals Screened for Prenatal Substance Use at Kaiser Permanente Northern California (KPNC) From 2015 to 2019 eFigure 2. Directed Acyclic Graph (DAG) Depicting Relationships Between Prenatal Cannabis Use, Confounders, and Early Developmental Delays eFigure 3. Hazard Ratios for Associations Between Prenatal Cannabis Use and Early Developmental Delays, Overall and by Frequency of Use, Among Pregnancies With No Other Prenatal Substance Use eFigure 4. Hazard Ratios for Associations Between Prenatal Cannabis Use and Early Developmental Delays, Overall and by Frequency of Use, Among Cohort With No Continuous Enrollment Requirement and Either Prenatal Substance Use Screening Questionnaire or Prenatal Urine Toxicology Test Completed eFigure 5. Cohort Assembly of Infants Born to Pregnant Individuals Screened for Prenatal Substance Use at Kaiser Permanente Northern California (KPNC) from 2015 to 2019, With No Continuous Enrollment Requirement and Either Prenatal Substance Use Screening Questionnaire or Prenatal Urine Toxicology Test Completed [file jamanetwopen-e2440295-s001.pdf]

Supplemental Online Content

Avalos LA, Oberman N, Alexeeff SE, et al. Early maternal prenatal cannabis use and child developmental delays. *JAMA Netw Open*. 2024;7(10):e2440295. doi:10.1001/jamanetworkopen.2024.40295

eAppendix 1. Ascertainment of Maternal Prenatal Cannabis Use and Other Substance Use

eAppendix 2. Ascertainment of Maternal Medical and Mental Health Comorbidities

eAppendix 3. Well-Child Visits and Censoring Intervals

eTable 1. Pregnancy Characteristics Stratified by Reported Frequency of Prenatal Cannabis Use

eTable 2. Hazard Ratios for Associations Between Prenatal Cannabis Use and Early Developmental Delays, Measured by Self-Report vs Urine Toxicology Results

eTable 3. Hazard Ratios for Associations Between Prenatal Cannabis Use Measured by Urine Toxicology Test and Early Developmental Delays, (n=119 976)

eFigure 1. Flow Diagram of Study Inclusion for Infants Born to Pregnant Individuals Screened for Prenatal Substance Use at Kaiser Permanente Northern California (KPNC) from 2015 to 2019

eFigure 2. Directed Acyclic Graph (DAG) Depicting Relationships Between Prenatal Cannabis Use, Confounders, and Early Developmental Delays

eFigure 3. Hazard Ratios for Associations Between Prenatal Cannabis Use and Early Developmental Delays, Overall and by Frequency of Use, Among Pregnancies with No Other Prenatal Substance Use

eFigure 4. Hazard Ratios for Associations Between Prenatal Cannabis Use and Early Developmental Delays, Overall and by Frequency of Use, Among Cohort With No Continuous Enrollment Requirement and Either Prenatal Substance Use Screening Questionnaire or Prenatal Urine Toxicology Test Completed

eFigure 5. Cohort Assembly of Infants Born to Pregnant Individuals Screened for Prenatal Substance Use at Kaiser Permanente Northern California (KPNC) from 2015 to 2019, With No Continuous Enrollment Requirement and Either Prenatal Substance Use Screening Questionnaire or Prenatal Urine Toxicology Test Completed

This supplemental material has been provided by the authors to give readers additional information about their work.

© 2024 Avalos LA et al. *JAMA Network Open.*

eAppendix 1. Ascertainment of Maternal Prenatal Cannabis Use and Other Substance Use

As part of standard prenatal care at KPNC, patients are universally screened for prenatal substance use by both self-report (via a self-administered questionnaire) and urine toxicology tests to which they provide consent at entrance into care. Of those individuals with continuous health plan membership in the year prior to pregnancy through delivery and attendance at a minimum of one prenatal care visit, 93.0% were screened by self-report and urine test for prenatal cannabis use (See eFigure 1).

Prenatal cannabis use was defined as self-reported cannabis use since becoming pregnant and/or a positive urine toxicology test at entrance to prenatal care. Results obtained from the first urine test during pregnancy ordered by OB/GYN department were used. If no urine test was ordered by OB/GYN department, results from the test closest to 8 weeks gestation were used. Cannabis toxicology screening tests were performed on a Beckman Coulter AU680 chemistry analyzer using the Emit II Plus Cannabinoid Assay from Siemens with a cutoff of 45ng/mL. Confirmatory testing for the presence of the cannabis metabolite, 11-nor-9-carboxy-delta 9- THC, was performed by liquid chromatography-tandem mass spectrometry for all positive immunoassay results. The confirmation test methodology was LC-MS/MS on a triple quadrupole system with a cutoff for positivity of 15ng/mL.

Other prenatal substance use was defined as the following:

Variable	Definition
Prenatal alcohol use	• Self-report for prenatal alcohol use at entrance to prenatal care<br>• Toxicology for ethanol at entrance to prenatal care[1]
Prenatal opioid use	• Self-report for prenatal heroin, methadone, or buprenorphine use at entrance to prenatal care<br>• Toxicology for 6-monoacetylmorphine (6-MAM, heroin), morphine, codeine, hydrocodone, norhydrocodone, hydromorphone, oxycodone, nor oxycodone, or oxymorphone at entrance to prenatal care[1]<br>• Opioid prescription filled during pregnancy before first prenatal visit date or before pregnancy with supply lasting past pregnancy start date
Prenatal stimulant use	• Self-report for prenatal cocaine, crack, or methamphetamine use at entrance to prenatal care<br>• Toxicology for amphetamine, methamphetamine, MDMA (3,4-methylenedioxymethamphetamine) or MDA (3,4-methylenedioxyamphetamine) at entrance to prenatal care[1]<br>• Amphetamine prescription filled during pregnancy before first prenatal visit date or before pregnancy with supply lasting past pregnancy start date
Prenatal nicotine use	• Self-report for prenatal nicotine use at entrance to prenatal care

© 2024 Avalos LA et al. *JAMA Network Open.*

	• Self-report for tobacco smoking during pregnancy before first prenatal visit date<br>• Nicotine replacement therapy prescription filled during pregnancy before first prenatal visit date or before pregnancy with supply lasting past pregnancy start date
Prenatal anxiety or sleep medication use	• Self-report for prenatal sleep or anxiety medication use at entrance to prenatal care<br>• Toxicology for benzodiazepines or barbiturates at entrance to prenatal care[1]<br>• Anxiolytics or sedative prescription filled during pregnancy before first prenatal visit date or before pregnancy with supply lasting past pregnancy start date

[1]Results from the first test urine test during pregnancy ordered by OB/GYN department. If no urine test was ordered by OB/GYN department, results from the test closest to 8 weeks gestation were used.

Self-reported prenatal substance use

Items from KPNC Early Start Prenatal Screening Questionnaire (revised 2014) used in this analysis:

In general, how often have you used the following **since pregnancy**:

1. Alcohol (wine, beer, liquor, etc.)
2. Nicotine (cigarettes, Nicorette gum, chewing tobacco, nicotine replacement therapy [NRT])
3. Sleep medication (Trazodone, Ambien, Restoril, etc.)
4. Pain medication (Vicodin, Norco, Oxycontin, Percocet, Codeine, etc.)
5. Anxiety medication (Valium, Xanax, Ativan, etc.)
6. Marijuana
7. Cocaine or Crack
8. Methamphetamine (Speed, Crank, Ecstasy, Ice, etc.)
9. Heroin
10. Other street drugs, please specify:
11. Methadone or Buprenorphine (Suboxone or Subutex)

Response options: Never, Monthly or Less, Weekly, Daily

Self-reported nicotine use was also assessed from EHR data on current tobacco use; KPNC patients are routinely asked about current tobacco use by medical assistants taking vital signs at the start of primary care visits.

Prenatal urine toxicology testing

Alcohol: Alcohol screening tests were performed on a Beckman Coulter AU680 chemistry analyzer using the Emit II Plus Ethyl Alcohol Assay from Siemens with a cutoff of 10 mg/dL.

© 2024 Avalos LA et al. *JAMA Network Open.*

Confirmatory testing for the presence of ethanol was performed by gas chromatography-mass spectrometry for all positive immunoassay results, also with a positive cutoff of 10 mg/dL.

Amphetamine/Methamphetamine: Amphetamine screening tests were performed on a Beckman Coulter AU680 chemistry analyzer using the DRI Amphetamines Assay with a cutoff of 500 ng/mL. Confirmatory testing for the presence of amphetamine species was performed by liquid chromatography- tandem mass spectrometry for all positive immunoassay results. Amphetamine species detected by the confirmation assay include amphetamine, methamphetamine, MDMA, and MDA. All species have a positive cutoff of 250 ng/mL.

Barbiturates: Barbiturate screening tests were performed on a Beckman Coulter AU680 chemistry analyzer using the Emit II Plus Barbiturate Assay from Siemens with a cutoff of 180 ng/mL. Confirmatory testing for the presence of barbiturate species was performed by liquid chromatography-tandem mass spectrometry for all positive immunoassay results. Barbiturate species detected by the confirmation assay include butalbital and phenobarbital. All species have a positive cutoff of 100 ng/mL.

Benzodiazepines: Benzodiazepine screening tests were performed on a Beckman Coulter AU680 chemistry analyzer using the CEDIA Benzodiazepine Assay from Thermo Fisher Scientific with a cutoff of 200 ng/mL. This assay incorporates beta-glucuronidase treatment to detect total benzodiazepine species for those that have glucuronidated metabolites. Confirmatory testing for the presence of benzodiazepine species was performed by liquid chromatography-tandem mass spectrometry for all positive immunoassay results. Benzodiazepine species detected by the confirmation assay include alprazolam/alpha-hydroxyalprazolam, clonazepam/7-aminoclonazepam, flunitrazepam/7-aminoflunitrazepam, lorazepam, nordiazepam, oxazepam, temazepam, alpha-hydroxytriazolam, and zolpidem. All species have a positive cutoff of 25 ng/mL except for zolpidem which has a positive cutoff of 2.5 ng/mL.

Cocaine: Cocaine screening tests were performed on a Beckman Coulter AU680 chemistry analyzer using the Emit II Plus Cocaine Metabolite Assay which detects the cocaine metabolite benzoylecgonine with a positive cutoff of 150 ng/mL. Confirmatory testing for the presence of benzoylecgonine was performed by liquid chromatography- tandem mass spectrometry for all positive immunoassay results. The positive cutoff for the confirmation assay was 100 ng/mL.

Opioids: Opiate screening tests were performed on a Beckman Coulter AU680 chemistry analyzer using the Emit II Plus Opiate 300 Assay from Siemens with a cutoff of 300 ng/mL. Confirmatory testing for the presence of opiate species was performed by liquid chromatography- tandem mass spectrometry for all positive immunoassay results. Opiate species detected by the confirmation assay include morphine, 6-monoacetylmorphine (6-MAM), codeine, hydrocodone, norhydrocodone, hydromorphone, oxycodone, noroxycodone, and oxymorphone. All species have a positive cutoff of 50 ng/mL except for 6-MAM which has a positive cutoff of 10 ng/mL.

© 2024 Avalos LA et al. *JAMA Network Open.*

Prescriptions filled during pregnancy

Class	Active Ingrdient
Antidepressants	AMITRIPTYLINE, AMITRIPTYLINE/CHLORDIAZEPOX, AMITRIPTYLINE/PERPHENAZINE, AMOXAPINE, BUPROPION, CITALOPRAM, CLOMIPRAMINE, DESIPRAMINE, DESVENLAFAXINE, DOXEPIN, DULOXETINE, ESCITALOPRAM, FLUOXETINE, FLUOXETINE/OLANZAPINE, FLUVOXAMINE, IMIPRAMINE, ISOCARBOXAZID, LEVOMILNACIPRAN, MAPROTILINE, MILNACIPRAN, MIRTAZAPINE, NEFAZODONE, NOMIFENSINE, NORTRIPTYLINE, PAROXETINE, PHENELZINE, PROTRIPTYLINE, SELEGILINE, SERTRALINE, TRANYLCYPROMINE, TRAZODONE, TRIMIPRAMINE, VENLAFAXINE, VILAZODONE, VORTIOXETINE
Opiates	BUPRENORPHINE, BUTALBITAL, BUTORPHANOL TARTRATE, CODEINE, DIHYDROCODEINE, FENTANYL, HYDROCODONE, HYDROMORPHONE, LEVORPHANOL TARTRATE, MEPERIDINE, METHADONE, MORPHINE SULFATE, MORPHINE SULFATE/NALTREXONE, OPIUM/BELLADONNA ALKALOIDS, OXYCODONE, OXYMORPHONE, PENTAZOCINE/ACETAMINOPHEN, PENTAZOCINE/ASPIRIN, PROPOXYPHENE, TAPENTADOL, TRAMADOL
ADHD medications	DEXMETHYLPHENIDATE, DEXTROAMPHETAMINE, DEXTROAMPHETAMINE/AMPHETAMINE, LISDEXAMFETAMINE, METHAMPHETAMINE, METHYLPHENIDATE, SERDEXMETHYLPHENIDATE/DEXMETHYLPHENIDATE
Sedatives	AMOBARBITAL, DEXMEDETOMIDINE, ESTAZOLAM, ESZOPICLONE, FLURAZEPAM, METHOHEXITAL, MIDAZOLAM, PENTOBARBITAL, QUAZEPAM, RAMELTEON, SCOPOLAMINE, SECOBARBITAL, SUVOREXANT, TASIMELTEON, TEMAZEPAM, TRIAZOLAM, ZALEPLON, ZOLPIDEM
Anxiolytics	BUSPIRONE, HYDROXYZINE, MEPROBAMATE
Nicotine Replacement	NICOTINE
Antiepileptics	METHYLPHENOBARBITAL, PHENOBARBITAL, PRIMIDONE, BARBEXACLONE, METHARBITA, ETHOTOIN, PHENYTOIN, AMINO(DIPHENYLHYDANTOIN) VALERIC ACID, MEPHENYTOIN, FOSPHENYTOIN, PHENYTOIN COMBINATIONS, MEPHENYTOIN COMBINATIONS, PARAMETHADIONE, TRIMETHADIONE, ETHADIONE, ETHOSUXIMIDE, PHENSUXIMIDE, MESUXIMIDE, ETHOSUXIMIDE COMBINATIONS, CLONAZEPAM, CARBAMAZEPINE, OXCARBAZEPINE, RUFINAMIDE, ESLICARBAZEPINE, VALPROIC ACID, VALPROMIDE, AMINOBUTYRIC ACID VIGABATRIN, PROGABIDE, TIAGABINE SULTIAME, PHENACEMIDE, LAMOTRIGINE, FELBAMATE, TOPIRAMATE, GABAPENTIN, PHENETURIDE, LEVETIRACETAM, ZONISAMIDE, PREGABALIN, STIRIPENTOL, LACOSAMIDE, CARISBAMATE, RETIGABINE, PERAMPANEL, BRIVARACETAM, CANNABIDIOL, BECLAMIDE
Teratogenics	ISOTRETINOIN, ACITRETIN, METHOTREXATE, MYCHOPHENOLATE, MISOPROSTOL, CARBIMAZOLE

© 2024 Avalos LA et al. *JAMA Network Open.*

Antineoplastics	CYCLOPHOSPHAMIDE, CHLORAMBUCIL, MELPHALAN, CHLORMETHINE, IFOSFAMIDE, TROFOSFAMIDE, PREDNIMUSTINE, BENDAMUSTINE, BUSULFAN, TREOSULFAN, MANNOSULFAN, THIOTEPA, TRIAZIQUONE, CARBOQUONE, CARMUSTINE, LOMUSTINE, SEMUSTINE, STREPTOZOCIN, FOTEMUSTINE, NIMUSTINE, RANIMUSTINE, URAMUSTINE, ETOGLUCID, MITOBRONITOL, PIPOBROMAN, TEMOZOLOMIDE, DACARBAZINE, METHOTREXATE , RALTITREXED, PEMETREXED, PRALATREXATE, MERCAPTOPURINE, TIOGUANINE, CLADRIBINE, FLUDARABINE, CLOFARABINE, NELARABINE, CYTARABINE, FLUOROURACIL, TEGAFUR, CARMOFUR, GEMCITABINE, CAPECITABINE, AZACITIDINE, DECITABINE, FLOXURIDINE, FLUOROURACIL, COMBINATIONS, TEGAFUR, COMBINATIONS, TRIFLURIDINE, COMBINATIONS, VINBLASTINE, VINCRISTINE, VINDESINE, VINORELBINE, VINFLUNINE, VINTAFOLIDE, ETOPOSIDE, TENIPOSIDE , DEMECOLCINE, PACLITAXEL, DOCETAXEL, PACLITAXEL POLIGLUMEX , CABAZITAXEL, TRABECTEDIN, DACTINOMYCIN, DOXORUBICIN, DAUNORUBICIN, EPIRUBICIN, ACLARUBICIN, ZORUBICIN, IDARUBICIN, MITOXANTRONE, PIRARUBICIN, VALRUBICIN, AMRUBICIN, PIXANTRONE, BLEOMYCIN, PLICAMYCIN, MITOMYCIN, IXABEPILONE, CISPLATIN, CARBOPLATIN, OXALIPLATIN, SATRAPLATIN, POLYPLATILLEN, PROCARBAZINE, EDRECOLOMAB, RITUXIMAB, TRASTUZUMAB, GEMTUZUMAB OZOGAMICIN, CETUXIMAB, BEVACIZUMAB, PANITUMUMAB, CATUMAXOMAB, OFATUMUMAB, IPILIMUMAB, BRENTUXIMAB VEDOTIN, PERTUZUMAB, TRASTUZUMAB EMTANSINE, OBINUTUZUMAB, DINUTUXIMAB BETA, NIVOLUMAB, PEMBROLIZUMAB, BLINATUMOMAB, RAMUCIRUMAB, NECITUMUMAB, ELOTUZUMAB, DARATUMUMAB, MOGAMULIZUMAB, INOTUZUMAB OZOGAMICIN, OLARATUMAB, DURVALUMAB, BERMEKIMAB, AVELUMAB, ATEZOLIZUMAB, CEMIPLIMAB, PORFIMER SODIUM, METHYL AMINOLEVULINATE, AMINOLEVULINIC ACID, TEMOPORFIN, EFAPROXIRAL, PADELIPORFIN, IMATINIB, GEFITINIB, ERLOTINIB, SUNITINIB, SORAFENIB, DASATINIB, LAPATINIB, NILOTINIB, TEMSIROLIMUS, EVEROLIMUS, PAZOPANIB, VANDETANIB, AFATINIB, BOSUTINIB, VEMURAFENIB, CRIZOTINIB, AXITINIB, RUXOLITINIB, RIDAFOROLIMUS, REGORAFENIB, MASITINIB, DABRAFENIB, PONATINIB, TRAMETINIB, CABOZANTINIB, IBRUTINIB, CERITINIB, LENVATINIB, NINTEDANIB, CEDIRANIB, PALBOCICLIB, TIVOZANIB, OSIMERTINIB, ALECTINIB, ROCILETINIB, COBIMETINIB, MIDOSTAURIN, OLMUTINIB, BINIMETINIB, RIBOCICLIB, BRIGATINIB, LORLATINIB, NERATINIB, ENCORAFENIB, DACOMITINIB, ICOTINIB, ABEMACICLIB, ACALABRUTINIB, QUIZARTINIB, LAROTRECTINIB, GILTERITINIB, ENTRECTINIB, FEDRATINIB, AMSACRINE, ASPARAGINASE, ALTRETAMINE, HYDROXYCARBAMIDE, LONIDAMINE, PENTOSTATIN,

© 2024 Avalos LA et al. *JAMA Network Open.*

	MASOPROCOL, ESTRAMUSTINE, TRETINOIN, MITOGUAZONE, TOPOTECAN, TIAZOFURINE, IRINOTECAN, ALITRETINOIN, MITOTANE, PEGASPARGASE, BEXAROTENE, ARSENIC TRIOXIDE, DENILEUKIN DIFTITOX, BORTEZOMIB, CELECOXIB, ANAGRELIDE, OBLIMERSEN, SITIMAGENE CERADENOVEC, VORINOSTAT, ROMIDEPSIN, OMACETAXINE MEPESUCCINATE, ERIBULIN, PANOBINOSTAT, VISMODEGIB, AFLIBERCEPT, CARFILZOMIB, OLAPARIB, IDELALISIB, SONIDEGIB, BELINOSTAT, IXAZOMIB, TALIMOGENE LAHERPAREPVEC, VENETOCLAX, VOSAROXIN, NIRAPARIB, RUCAPARIB, ETIRINOTECAN PEGOL, PLITIDEPSIN, EPACADOSTAT, ENASIDENIB, TALAZOPARIB, COPANLISIB, IVOSIDENIB, GLASDEGIB, ENTINOSTAT, ALPELISIB, SELINEXOR, TAGRAXOFUSP, BELOTECAN, CYTARABINE, DAUNORUBICIN

© 2024 Avalos LA et al. *JAMA Network Open.*

eAppendix 2. Ascertainment of Maternal Medical and Mental Health Comorbidities

Comorbidity	ICD-9 and ICD-10 Diagnosis Codes
Asthma	ICD-9: 493.*, 495.8, 518.3, 518.81-518.84, 519.11, 786.07<br>ICD-10: J45.*, J82.83, J82.89, J96.*, J98.01, R06.2
Diabetes mellitus	Identified with the KPNC Diabetes Registry which is updated annually using a validated algorithm to identify KPNC members with diabetes. See: Weiner JZ, Gopalan A, Mishra P, et al. Use and Discontinuation of Insulin Treatment Among Adults Aged 75 to 79 Years with Type 2 Diabetes. JAMA Intern Med. 2019;179(12):1633–1641. doi:10.1001/ jamainternmed.2019. 3759.
Nausea and vomiting in pregnancy	ICD-9: 536.2, 643.*, 787.01 - 787.04,<br>ICD-10: G43.A0, G43.A1, O21.*, R11.*
Mood/anxiety disorders	*Anxiety*<br>ICD-9: 293.84, 300.02, 300.09, 309.24, 648.4<br>ICD-10: F06.4, F41.1, F41.3, F41.8, F41.9, F43.22<br>*Bipolar disorder*<br>ICD-9: 296.0* (except 296.06) 296.1* (except 296.16), 296.4* (except 296.46), 296.5* (except 296.56), 296.6* (except 296.66), 301.13, 309.28<br>ICD-10: F06.33, F30.* (except F30.4), F31.* (except F31.70, F31.72, F31.74, F31.76, F31.78), F34.0<br>*Depression*<br>ICD-9: 296.2X (except 296.26), 296.3* (except 296.36), 296.82, 300.4, 309.0, 309.1, 311, 648.4*<br>ICD-10: F06.31, F06.32, F32.0, F32.1, F32.2, F32.3, F32.4, F32.8, F32.81, F32.89, F32.9, F33.0, F33.1, F33.2, F33.3, F33.41, F33.8, F33.9, F34.1, F43.21, F43.23, F53.0, O90.6, O99.34*
Other psychiatric disorders	*ADHD*<br>ICD-9: 314.*<br>ICD-10: F90.*<br>*Adjustment disorder*<br>ICD-9: 309.22, 309.23, 309.29, 309.3, 309.4, 309.8X, 309.9, 313.*<br>ICD-10: F43.2* (except F43.23)<br>*Alcohol-induced disorders*<br>ICD-9: 291.*<br>ICD-10: F10.121, F10.131, F10.14, F10.15*, F10.18* (except F10.181), F10.19, F10.221, F10.231, F10.232, F10.24, F10.25*, F10.26, F10.27, F10.28* (except F10.281), F10.29, F10.98* (except F10.981), F10.99<br>*Conduct disorder*<br>ICD-9: 312.*, 313.81<br>ICD-10: F91.*<br>*Dementia*<br>ICD-9: 290.*, 294.1*, 294.2*, 310.8*, 331.*, 780.93, 780.97<br>ICD-10: F01.5*, F02.8*, F03.*, F48.8, G30.*, G31.84, R41.82<br>*Drug-induced disorders*<br>ICD-9: 292.*<br>ICD-10: F11.121, F11.122, F11.14, F11.15*, F11.18* (except F11.181), F11.19, F11.221, F11.222, F11.24, F11.25*, F11.28* (except F11.281), F11.29, F11.921, F11.922, F11.94, F11.95*, F11.98* (except F11.981); F12.121, F12.122, F12.15*, F12.18*, F12.19, F12.221, F12.222, F12.25*, F12.28* F12.29, F12.921, F12.922, F12.95*, F12.98*, F12.99; F13.132, F13.15*, F13.232, F13.25*, F13.932, F13.95*;

© 2024 Avalos LA et al. *JAMA Network Open.*

	F14.121, F14.122, F14.14, F14.15*, F14.18* (except F14.181), F14.19, F14.221, F14.222, F14.24, F14.25*, F14.28* (except F14.281), F14.29, F14.921, F14.922, F14.94, F14.95*, F14.98* (except F14.981);<br>F15.121, F15.122, F15.14, F15.15*, F15.18* (except F15.181), F15.19, F15.221, F15.222, F15.24, F15.25*, F15.28* (except F15.281), F15.29, F15.921, F15.922, F15.94, F15.95*, F15.98* (except F15.981);<br>F16.121, F16.122, F16.14, F16.15*, F16.18*, F16.19, F16.221, F16.24, F16.25*, F16.28*, F16.29, F16.921, F16.94, F16.95*, F16.98*, F16.99;<br>F17.208, F17.209, F17.218, F17.219, F17.228, F17.229, F17.298, F17.299;<br>F18.121, F18.14, F18.15*, F18.17, F18.18*, F18.19, F18.221, F18.24, F18.25*, F18.27, F18.28*, F18.29, F18.921, F18.94, F18.95*, F18.97, F18. F18.98*, F18.99;<br>F19.121, F19.122, F19.131, F19.132, F19.14, F19.15*, F19.16, F19.17, F19.18*, F19.19, F19.221, F19.222, F19.231, F19.232, F19.24, F19.25*, F19.26, F19.27, F19.28*, F19.29, F19.921, F19.922, F19.931, F19.932, F19.94, F19.95*, F19.96, F19.97, F19.98*, F19.99;<br>*Eating disorders*<br>ICD-9: 307.1, 307.5*<br>ICD-10: F50.*, R63.0<br>*Other psychological disorders*<br>ICD-9: 297.*, 298.* 300.5, 300.6, 300.7, 300.8*, 307.3, 307.5, 316<br>ICD-10: F21, F22, F23, F24, F28, F29, F53.1<br>*Personality disorders*<br>ICD-9: 301.* (except 301.13)<br>ICD-10: F60.*<br>*Schizophrenia*<br>ICD-9: 295.*<br>ICD-10: F20.*<br>*Suicide and self-inflicted injury*<br>ICD-9: E950.*–E958.*, E980.*-E988.*<br>ICD-10: T14.91*, T36.* – T65.*, T71.*, X71.*-X83.*, Y21.*-Y33.*<br>*Tic disorders*<br>ICD-9: 307.2*<br>ICD-10: F95.*
Substance use disorders (except cannabis use disorders)	ICD-9: 303.* (except 303.03, 303.93), 304.* (except 304.03, 304.13, 304.23, 304.3*, 304.43, 304.53, 304.63,304.73, 304.83, 304.93), 305.* (except 305.03, 305.13, 305.2*, 305.33, 305.43, 305.53, 305.63, 305.73, 305.83, 305.93)<br>ICD-10: F10.1, F10.10, F10.12* (except F10.121) F10.13* (except F10.131, F10.132), F10.181, F10.2, F10.20, F10.22* (except F10.221) F10.23* (except F10.231, F10.232), F10.281, F10.929, F10.93*(except F10.931, F10.932) F10.981;<br>F11.1, F11.10, F11.12* (except F11.121, F11.122) F11.13, F11.181, F11.2, F11.20, F11.22* (except F11.221, F11.222), F11.23, F11.281, F11.9, F11.90, F11.92* (except F11.921, F11.922), F11.93, F11.981;<br>F13.1, F13.10, F13.12*, F13.13* (except F13.132), F13.20, F13.22*, F13.23*, F13.9, F13.90, F13.92*, F13.930;<br>F14.1, F14.10, F14.12* (except F14.121, F14.122) F14.13, F14.181, F14.2, F14.20, F14.22* (except F14.221, F14.222), F14.23, F14.281, F14.9, F14.90, F14.92* (except F14.921, F14.922), F14.93, F14.981;

© 2024 Avalos LA et al. *JAMA Network Open.*

	F15.1, F15.10, F15.12* (except F15.121, F15.122) F15.13, F15.181, F15.2, F15.20, F15.22* (except F15.221, F15.222), F15.23, F15.281, F15.9, F15.90, F15.92* (except F15.921, F15.922), F15.93, F15.981;<br>F16.1, F16.10, F16.12* (except F16.121, F16.122) F16.2, F16.20, F16.22* (except F16.221), F16.9, F16.90, F16.92* (except F16.921);<br>F17.2, F17.20* (except F17.208, F17.209), F17.21* (except F17.218, F17.219) F17.22* (except F17.228, F17.229), F17.29* (except F17.298, F17.299);<br>F18.1, F18.10, F18.12* (except F18.121) F18.2, F18.20, F18.22* (except F18.221), F18.9, F18.90, F18.92* (except F18.921);<br>F19.1, F19.10, F19.12* (except F19.121, F19.122) F19.13* (except F19.131, F19.132), F19.181, F19.2, F19.20, F19.22* (except F19.221, F19.222), F19.23* (Except F19.231, F19.232), F19.281, F19.9, F19.90, F19.92* (except F19.921, F19.922), F19.93*(except F19.931, F19.932), F19.981;<br>Z71.6
Chronic pain	ICD-9: 307.8*, 307.2, 338.*<br>ICD-10: G89.*, F45.4*

eAppendix 3. Well-child Visits and Censoring Intervals

Well-child visits were defined as an encounter labeled with KPNC-specific codes for a well-child visit or physical exam with well-child care. Well-child visits were categorized by calculating the child's age on the encounter date based on the number of months from their birth date as follows: 2-month visit: ≥1 to < 3 months old, 4-month visit: ≥3 to <5 months old, 6-month visit: ≥5 to <10 months old, 12-month visit: ≥10 to <15 months old, 18-month visit: ≥15 to <20 months old, 24-month visit: ≥20 to <28 months old, 3-year visit: ≥28 to 42 months old, 4-5 year visit: ≥ 42 to 66 months.

Children were censored based on the following criteria:

**Well-Child visit**	**Time interval**	**Censoring criteria**
2 months	≥1 month to <3months	At least 1 required. If none, censor at 10 months
4 months	≥3 months to <5months	
6 months	≥5 months to <10 months	
12 months	≥10 months to < 15 months	Required. If none, censor at 15 months
18 months	≥15 months to < 20 months	At least 1 required. If none, censor at 28 months
21 to 24 months	≥20 months to <28 months	
3 years	≥28 months to <42 months	Required. If none, censor at 42 months
4-5 years	≥42 months to 66 months	

eTable 1. Pregnancy Characteristics Stratified by Reported Frequency of Prenatal Cannabis Use

	Frequency of Prenatal Cannabis Use				
**Characteristic**	**Never, N (%)**	**Monthly or less, N (%)**	**Weekly, N (%)**	**Daily, N (%)**	**Unknown Frequency[a], N (%)**
**Total pregnancies[b]**	113,198	1,617	722	618	3,821
**Maternal sociodemographic characteristics**					
Race/ethnicity					
Non-Hispanic White	44,209 (39.1)	617 (38.2)	306 (42.4)	237 (38.3)	1,454 (38.1)
Asian/Pacific Islander	32,334 (28.6)	161 (10.0)	49 (6.8)	26 (4.2)	223 (5.8)
Hispanic	27,600 (24.4)	456 (28.2)	171 (23.7)	153 (24.8)	1,163 (30.4)
Non-Hispanic Black	5,218 (4.6)	282 (17.4)	150 (20.8)	166 (26.9)	751 (19.7)
Other[c]/Unknown	3,837 (3.4)	101 (6.2)	46 (6.4)	36 (5.8)	230 (6.0)
Age at pregnancy onset					
<18	466 (0.4)	41 (2.5)	14 (1.9)	10 (1.6)	66 (1.7)
18-24	10,046 (8.9)	475 (29.4)	248 (34.3)	234 (37.9)	1,237 (32.4)
25-30	36,404 (32.2)	513 (31.7)	224 (31.0)	213 (34.5)	1,267 (33.2)
31-35	44,440 (39.3)	416 (25.7)	162 (22.4)	112 (18.1)	863 (22.6)
36+	21,842 (19.3)	172 (10.6)	74 (10.2)	49 (7.9)	388 (10.2)
Parity					
0	46,123 (40.7)	961 (59.4)	441 (61.1)	374 (60.5)	1,895 (49.6)
1	42,710 (37.7)	388 (24.0)	180 (24.9)	136 (22.0)	1,200 (31.4)
2+	24,365 (21.5)	268 (16.6)	101 (14.0)	108 (17.5)	726 (19.0)
Insurance type					
Medicaid	8,641 (7.6)	374 (23.1)	184 (25.5)	196 (31.7)	970 (25.4)
Non-Medicaid	104,557 (92.4)	1,243 (76.9)	538 (74.5)	422 (68.3)	2,851 (74.6)
Education level					
High School or Less	13,869 (12.3)	384 (23.7)	215 (29.8)	204 (33.0)	1,170 (30.6)
Some College	31,644 (28.0)	698 (43.2)	318 (44.0)	297 (48.1)	1,774 (46.4)
College Graduate	39,303 (34.7)	347 (21.5)	126 (17.5)	77 (12.5)	555 (14.5)
Graduate School	25,845 (22.8)	150 (9.3)	53 (7.3)	24 (3.9)	219 (5.7)
Unknown	2,537 (2.2)	38 (2.4)	10 (1.4)	16 (2.6)	103 (2.7)
Neighborhood Deprivation Index					
Q1 - Least deprived	29,096 (25.7)	295 (18.2)	115 (15.9)	63 (10.2)	419 (11.0)
Q2	28,662 (25.3)	354 (21.9)	141 (19.5)	109 (17.6)	734 (19.2)
Q3	28,174 (24.9)	416 (25.7)	187 (25.9)	153 (24.8)	1,072 (28.1)
Q4 - Most deprived	27,266 (24.1)	552 (34.1)	279 (38.6)	293 (47.4)	1,596 (41.8)

© 2024 Avalos LA et al. *JAMA Network Open.*

**Other (non-cannabis) prenatal substance use[d]**					
Alcohol use	9,741 (8.6)	728 (45.0)	267 (37.0)	219 (35.4)	152 (4.0)
Nicotine use	2,825 (2.5)	332 (20.5)	214 (29.6)	216 (35.0)	540 (14.1)
Anxiety/Sleep medication use	2,853 (2.5)	140 (8.7)	80 (11.1)	66 (10.7)	197 (5.2)
Stimulant use	659 (0.6)	76 (4.7)	37 (5.1)	36 (5.8)	66 (1.7)
Opioid use	2,100 (1.9)	83 (5.1)	48 (6.6)	45 (7.3)	198 (5.2)
**Prenatal care utilization**					
Kotelchuck month of initiation index					
Inadequate (Month 7+)	371 (0.3)	27 (1.7)	8 (1.1)	15 (2.4)	14 (0.4)
Intermediate (Month 5-6)	1,073 (0.9)	48 (3.0)	22 (3.0)	13 (2.1)	53 (1.4)
Adequate (Month 3-4)	30,140 (26.6)	507 (31.4)	241 (33.4)	164 (26.5)	980 (25.6)
Adequate Plus (Month 1-2)	81,614 (72.1)	1,035 (64.0)	451 (62.5)	426 (68.9)	2,774 (72.6)
**Maternal comorbidities**					
Diabetes (Type I or II)[e]	1,642 (1.5)	16 (1.0)	6 (0.8)	10 (1.6)	54 (1.4)
Asthma[f]	11,832 (10.5)	278 (17.2)	154 (21.3)	139 (22.5)	690 (18.1)
Nausea/vomiting in pregnancy[g]	11,586 (10.2)	308 (19.0)	177 (24.5)	150 (24.3)	979 (25.6)
Mood/anxiety disorders[f]	14,018 (12.4)	423 (26.2)	213 (29.5)	201 (32.5)	937 (24.5)
Other psychiatric disorders[f]	2,782 (2.5)	132 (8.2)	61 (8.4)	50 (8.1)	243 (6.4)
Antidepressant use[h]	4,978 (4.4)	132 (8.2)	75 (10.4)	87 (14.1)	336 (8.8)
Chronic pain[f]	4,038 (3.6)	100 (6.2)	53 (7.3)	62 (10.0)	271 (7.1)
Substance use disorders[f,i]	2,709 (2.4)	231 (14.3)	162 (22.4)	164 (26.5)	503 (13.2)

[a]Positive urine toxicology test for Tetrahydrocannabinol (THC), but reported no cannabis use since becoming pregnant

[b]The cohort consisted of 119,976 unique pregnancies in 106,240 unique pregnant individuals.

[c]Other race/ethnicity includes American Indian, Alaskan Native, and multi-racial individuals.

[d]Assessed at entrance to prenatal care or prescription filled during pregnancy through date of first prenatal visit. See eAppendix 2 for full details.

[e]In two years prior to pregnancy onset

[f]In year prior to pregnancy onset or during pregnancy through date of first prenatal visit

[g]Through date of first prenatal visit

[h]Prescription fill before pregnancy with supply lasting through pregnancy onset or during pregnancy through date of first prenatal visit

[i]Excludes cannabis-related disorders

© 2024 Avalos LA et al. *JAMA Network Open.*

eTable 2. Hazard Ratios for Associations Between Prenatal Cannabis Use Measured by Self-Report and Early Developmental Delays, n=119,976

	No. Events	aHR[a] (95% CI)
**Speech and Language Disorders**		
Self-Reported Prenatal Cannabis Use		
No	8,447	1.00 (Ref)
Yes	184	0.97 (0.83 - 1.14)
Frequency of Self-Reported Prenatal Cannabis Use		
None	8,447	1.00 (Ref)
Monthly or less	98	0.96 (0.78 - 1.17)
Weekly	43	0.92 (0.67 - 1.26)
Daily	43	1.08 (0.79 - 1.48)
**Global Delay**		
Self-Reported Prenatal Cannabis Use		
No	428	1.00 (Ref)
Yes	11	0.81 (0.42 - 1.58)
Frequency of Self-Reported Prenatal Cannabis Use		
None	428	1.00 (Ref)
Monthly or less	4	0.59 (0.21 - 1.63)
Weekly	4	1.08 (0.39 - 3.03)
Daily	3	1.00 (0.30 - 3.33)
**Motor Delay**		
Self-Reported Prenatal Cannabis Use		
No	2,095	1.00 (Ref)
Yes	41	0.83 (0.60 - 1.14)
Frequency of Self-Reported Prenatal Cannabis Use		
None	2,095	1.00 (Ref)
Monthly or less	20	0.73 (0.47 - 1.14)
Weekly	11	0.84 (0.46 - 1.54)
Daily	10	1.06 (0.56 - 2.02)

aHR= Adjusted Hazard Ratio; CI= Confidence Interval

[a]Adjusted for sociodemographic variables, other non-cannabis substance use, prenatal care initiation, and medical and mental health comorbidities.

© 2024 Avalos LA et al. *JAMA Network Open.*

eTable 3. Hazard Ratios for Associations Between Prenatal Cannabis Use Measured by Urine Toxicology Test and Early Developmental Delays, n=119,976

	No. Events	aHR[a] (95% CI)
**Speech and Language Disorders**		
Prenatal Cannabis Use by Urine Toxicology		
No	8,305	1.00 (Ref)
Yes	326	0.88 (0.78 - 0.99)
**Global Delay**		
Prenatal Cannabis Use by Urine Toxicology		
No	415	1.00 (Ref)
Yes	24	1.05 (0.66 - 1.67)
**Motor Delay**		
Prenatal Cannabis Use by Urine Toxicology		
No	2,060	1.00 (Ref)
Yes	76	0.85 (0.67 - 1.08)

aHR= Adjusted Hazard Ratio; CI= Confidence Interval

[a]Adjusted for maternal sociodemographic characteristics, other non-cannabis substance use, prenatal care initiation, and medical and mental health comorbidities.

© 2024 Avalos LA et al. *JAMA Network Open.*

eFigure 1. Cohort assembly of infants born to pregnant individuals screened for substance use in pregnancy at Kaiser Permanente Northern California (KPNC) from 2015 to 2019

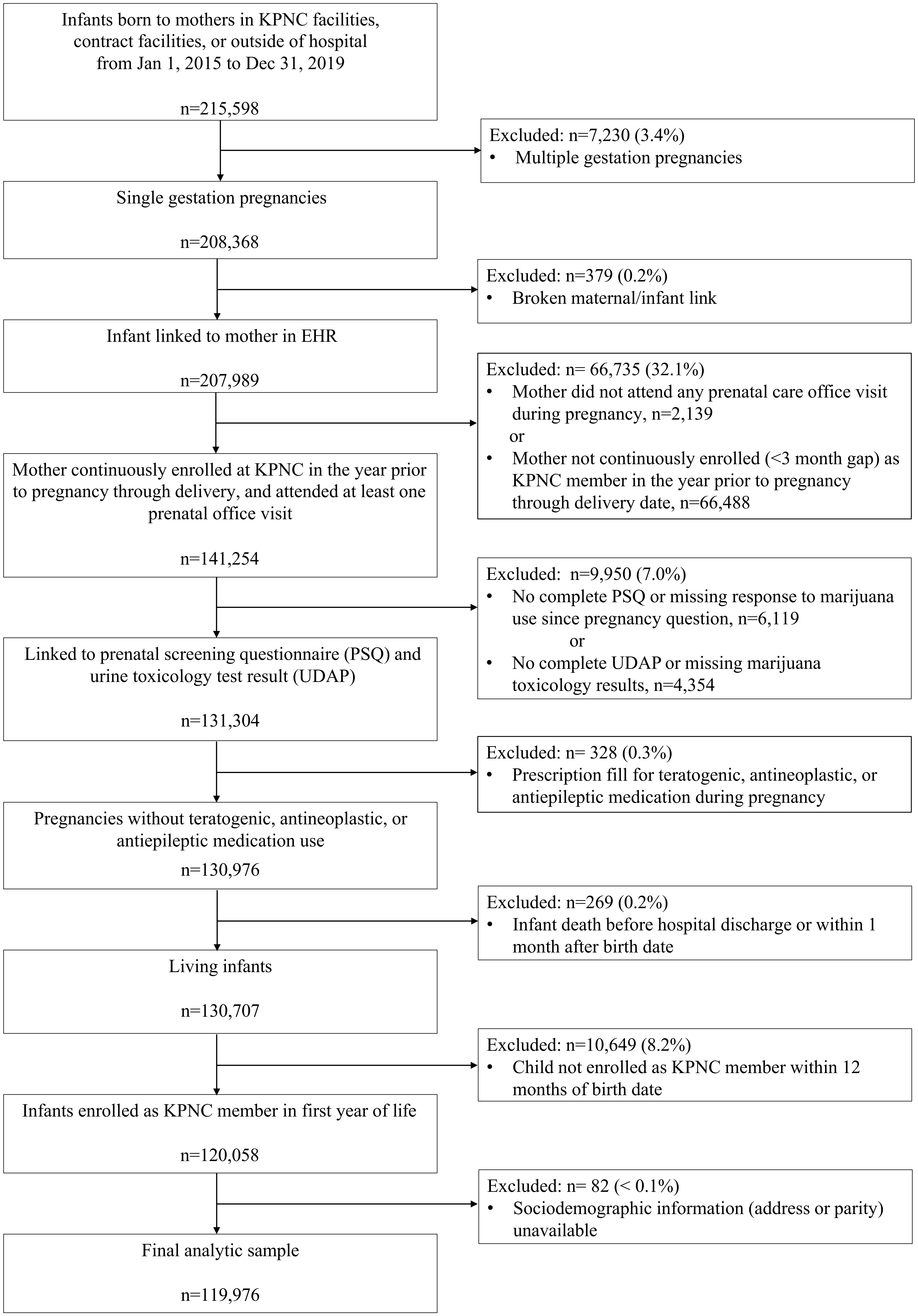

© 2024 Avalos LA et al. *JAMA Network Open.*

eFigure 2. Directed Acyclic Graph (DAG) Depicting Relationships Between Prenatal Cannabis Use, Confounders, and Early Developmental Delays

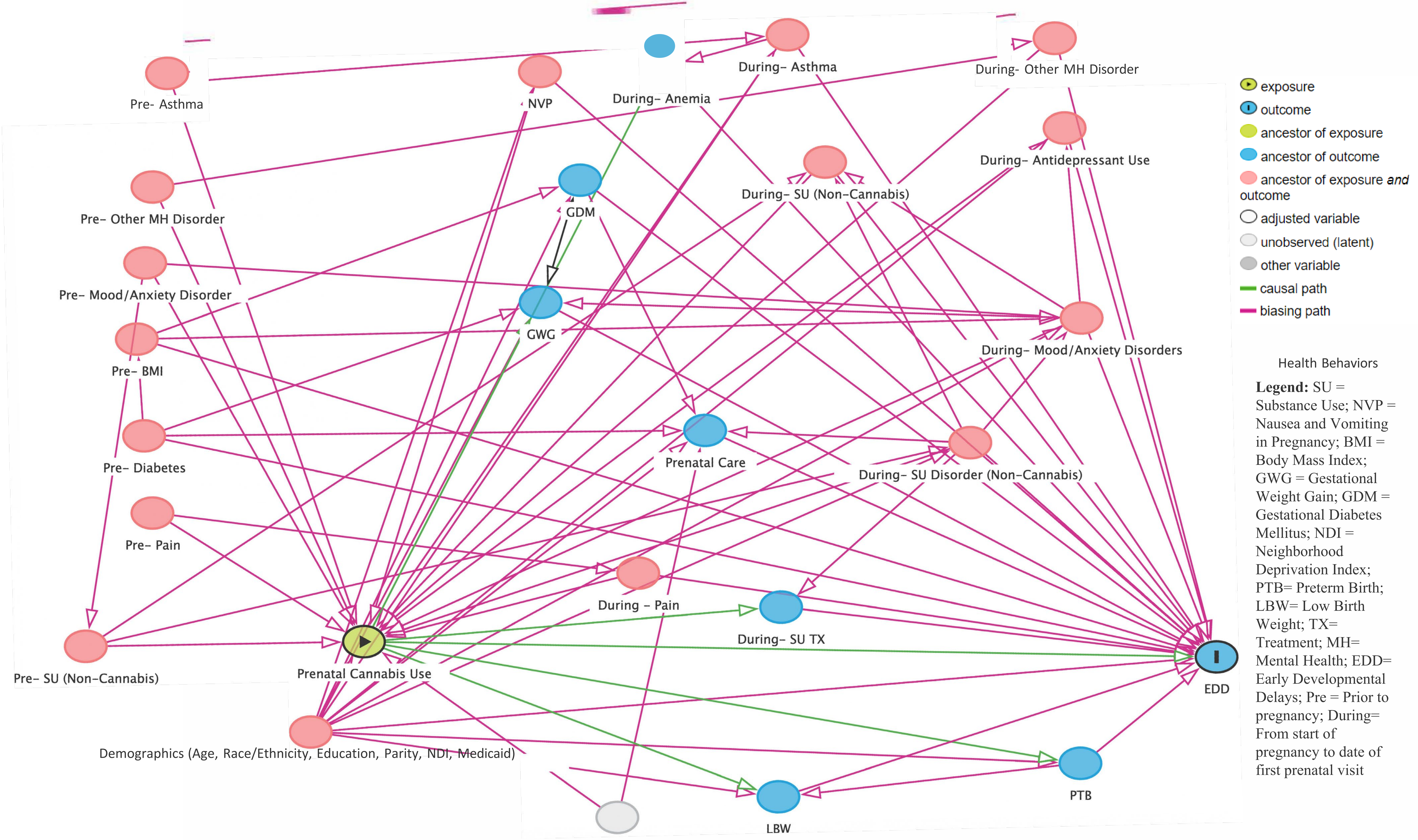

Legend: SU = Substance Use; NVP = Nausea and Vomiting in Pregnancy; BMI = Body Mass Index; GWG = Gestational Weight Gain; GDM = Gestational Diabetes Mellitus; NDI = Neighborhood Deprivation Index; PTB= Preterm Birth; LBW= Low Birth Weight; TX= Treatment; MH= Mental Health; EDD= Early Developmental Delays; Pre = Prior to pregnancy; During= From start of pregnancy to date of first prenatal visit

© 2024 Avalos LA et al. *JAMA*

Minimal sufficient adjustment sets for estimating the total effect of in utero cannabis on development:

- During -Other Psych, During Asthma, GDM, NVP, Pre- BMI, Pre- diabetes, demographics (age, race/ethnicity, education, parity, NDI, Medicaid), during - Pain, during SU (not cannabis), during SU disorder, during antidepressant, during mood/anxiety disorders, prenatal care
- During -Other Psych, During Asthma, GDM, NVP, Pre- diabetes, Pre-Mood/anxiety dis, demographics (age, race/ethnicity, education, parity, NDI, Medicaid), during - Pain, during SU (not cannabis), during SU disorder, during antidepressant, during mood/anxiety disorders, prenatal care

Daggity Code:

```
dag {
"During -Other Psych" [pos="0.290,-2.547"]
"During Asthma" [pos="-0.421,-2.630"]
"Health Behaviors" [latent,pos="-0.869,2.372"]
"Pre- BMI" [pos="-2.406,-0.820"]
"Pre- Pain" [pos="-2.375,0.133"]
"Pre- SU" [latent,pos="-2.628,2.389"]
"Pre- diabetes" [pos="-2.406,-0.298"]
"Pre- other psych disorder" [pos="-2.422,-1.789"]
"Pre-Mood/anxiety dis" [pos="-2.406,-1.366"]
"Pre-SU" [pos="-2.392,1.305"]
"Pre-asthma"  [pos="-2.428,-2.670"]
"demographics (age, race/ethnicity, education, parity, NDI, Medicaid)" [pos="-1.544,2.027"]
"depression severity" [latent,pos="1.194,-2.009"]
"dur anemia" [pos="-0.753,-2.427"]
"dur-SU tx" [pos="-0.438,1.028"]
"during - Pain" [pos="-0.814,0.811"]
"during SU (not can)" [pos="-0.169,-1.773"]
"during SU disorder" [pos="0.060,-0.023"]
"during antidepressant" [pos="0.264,-1.917"]
"during mood/anxiety disorders" [pos="0.353,-0.820"]
"in utero cannabis" [exposure,pos="-1.536,1.249"]
"prenatal care" [pos="-0.638,-0.101"]
GDM [pos="-0.967,-1.701"]
GWG [pos="-1.071,-0.920"]
LBW [pos="-0.445,2.240"]
NVP [pos="-1.073,-2.395"]
PTB [pos="0.350,2.026"]
development [outcome,pos="1.197,1.297"]
"During -Other Psych" -> "in utero cannabis"
"During -Other Psych" -> development
"During Asthma" -> "in utero cannabis"
"During Asthma" -> development
"Health Behaviors" -> "in utero cannabis"
"Health Behaviors" -> "prenatal care"
"Pre- BMI" -> "during mood/anxiety disorders"
"Pre- BMI" -> GDM
"Pre- BMI" -> development
"Pre- Pain" -> "during - Pain"
"Pre- Pain" -> "in utero cannabis"
"Pre- diabetes" -> "Pre- BMI"
"Pre- diabetes" -> "prenatal care"
"Pre- diabetes" -> GWG
"Pre- diabetes" -> development
"Pre- other psych disorder" -> "During -Other Psych"
"Pre- other psych disorder" -> "in utero cannabis"
```

© 2024 Avalos LA et al. *JAMA Network Open.*

```
"Pre-Mood/anxiety dis" -> "Pre-SU"
"Pre-Mood/anxiety dis" -> "during mood/anxiety disorders"
"Pre-Mood/anxiety dis" -> "in utero cannabis"
"Pre-SU" -> "during SU (not can)"
"Pre-SU" -> "during SU disorder"
"Pre-SU" -> "in utero cannabis"
"Pre-asthma" -> "During Asthma"
"Pre-asthma" -> "in utero cannabis"
"demographics (age, race/ethnicity, education, parity, NDI, Medicaid)" -> "During Asthma"
"demographics (age, race/ethnicity, education, parity, NDI, Medicaid)" -> "during SU disorder"
"demographics (age, race/ethnicity, education, parity, NDI, Medicaid)" -> "during antidepressant"
"demographics (age, race/ethnicity, education, parity, NDI, Medicaid)" -> "during mood/anxiety disorders"
"demographics (age, race/ethnicity, education, parity, NDI, Medicaid)" -> "in utero cannabis"
"demographics (age, race/ethnicity, education, parity, NDI, Medicaid)" -> "prenatal care"
"demographics (age, race/ethnicity, education, parity, NDI, Medicaid)" -> GDM
"demographics (age, race/ethnicity, education, parity, NDI, Medicaid)" -> GWG
"demographics (age, race/ethnicity, education, parity, NDI, Medicaid)" -> LBW
"demographics (age, race/ethnicity, education, parity, NDI, Medicaid)" -> NVP
"demographics (age, race/ethnicity, education, parity, NDI, Medicaid)" -> PTB
"demographics (age, race/ethnicity, education, parity, NDI, Medicaid)" -> development
"dur anemia" -> development
"dur-SU tx" -> development
"during - Pain" -> "in utero cannabis"
"during - Pain" -> development
"during SU (not can)" -> "in utero cannabis"
"during SU (not can)" -> development [pos="0.993,1.333"]
"during SU disorder" -> "dur-SU tx"
"during SU disorder" -> "during SU (not can)"
"during SU disorder" -> "during mood/anxiety disorders"
"during SU disorder" -> "in utero cannabis"
"during SU disorder" -> "prenatal care"
"during SU disorder" -> development
"during antidepressant" -> "in utero cannabis"
"during antidepressant" -> development
"during mood/anxiety disorders" -> "during SU (not can)"
"during mood/anxiety disorders" -> "during antidepressant"
"during mood/anxiety disorders" -> "in utero cannabis"
"during mood/anxiety disorders" -> GWG
"during mood/anxiety disorders" -> development
"in utero cannabis" -> "dur anemia"
"in utero cannabis" -> "dur-SU tx"
"in utero cannabis" -> GWG
"in utero cannabis" -> LBW
"in utero cannabis" -> PTB
"in utero cannabis" -> development
"prenatal care" -> development
GDM -> "prenatal care"
GDM -> development
GDM <-> GWG
GWG -> development
LBW -> development
NVP -> "in utero cannabis"
NVP -> development
PTB -> LBW
PTB -> development
}
```

© 2024 Avalos LA et al. *JAMA Network Open.*

eFigure 3. Hazard Ratios for Associations Between Prenatal Cannabis Use and Early Developmental Delays, Overall and by Frequency of Use Among Pregnancies with No Other Prenatal Substance Use; n=101,011

	No. Events	aHR[a] (95% CI)
**Speech and Language Disorders**		
Prenatal Cannabis Use		
No	7,019	1.00 (Ref)
Yes	232	0.89 (0.77 − 1.02)
Frequency of Prenatal Cannabis Use		
None	7,019	1.00 (Ref)
Monthly or Less	27	0.68 (0.46 − 1.00)
Weekly	11	0.65 (0.36 − 1.19)
Daily	16	1.08 (0.65 − 1.79)
Unknown Frequency[b]	178	0.93 (0.80 − 1.09)
**Global Delay**		
Prenatal Cannabis Use		
No	347	1.00 (Ref)
Yes	15	0.97 (0.57 − 1.67)
Frequency of Prenatal Cannabis Use		
None	347	1.00 (Ref)
Monthly or Less	1	0.43 (0.06 − 3.19)
Weekly	1	0.82 (0.11 − 5.89)
Daily	3	2.45 (0.83 − 7.23)
Unknown Frequency[b]	10	0.94 (0.49 − 1.81)
**Motor Delay**		
Prenatal Cannabis Use		
No	1,709	1.00 (Ref)
Yes	51	0.78 (0.59 − 1.04)
Frequency of Prenatal Cannabis Use		
None	1,709	1.00 (Ref)
Monthly or Less	5	0.49 (0.20 − 1.18)
Weekly	3	0.64 (0.20 − 2.02)
Daily	5	1.33 (0.55 − 3.24)
Unknown Frequency[b]	38	0.82 (0.59 − 1.13)

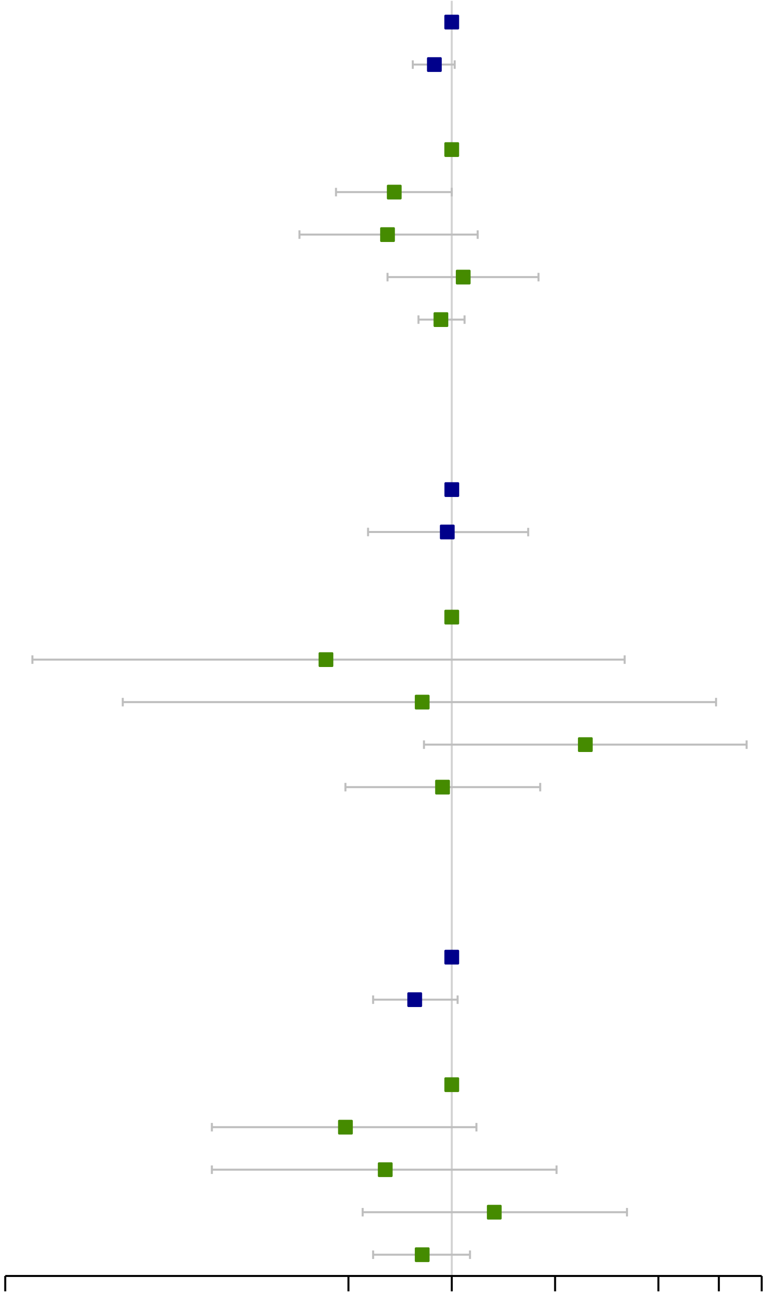

aHR= Adjusted Hazard Ratio; CI= Confidence Interval

aHR (log scale)

0.05 0.50 1.00 2.00 4.00 6.00

[a]Adjusted for maternal sociodemographic characteristics, prenatal care initiation, and medical and mental health comorbidities
[b]Positive urine toxicology test for Tetrahydrocannabinol (THC), but reported no cannabis use since becoming pregnant

© 2024 Avalos LA et al. *JAMA Network Open.*

eFigure 4. Hazard Ratios for Associations Between Prenatal Cannabis Use and Early Developmental Delays, Overall and by Frequency of Use, Among Cohort With No Continuous Enrollment Requirement and Either Prenatal Substance Use Screening Questionnaire or Prenatal Urine Toxicology Test Completed; n=181,700

	No. Events	aHR[a] 95% CI
**Speech and Language Disorders**		
Prenatal Cannabis Use		
No	12,162	1.00 (Ref)
Yes	602	0.93 (0.85 – 1.02)
Frequency of Prenatal Cannabis Use		
None	12,162	1.00 (Ref)
Monthly or Less	138	0.93 (0.78 – 1.10)
Weekly	57	0.83 (0.63 – 1.09)
Daily	61	0.99 (0.76 – 1.29)
Unknown Frequency[b]	346	0.94 (0.84 – 1.06)
**Global Delay**		
Prenatal Cannabis Use		
No	577	1.00 (Ref)
Yes	38	0.88 (0.71 – 1.09)
Frequency of Prenatal Cannabis Use		
None	577	1.00 (Ref)
Monthly or Less	6	0.90 (0.59 – 1.37)
Weekly	5	0.52 (0.26 – 1.07)
Daily	3	0.59 (0.29 – 1.22)
Unknown Frequency[b]	24	0.98 (0.75 – 1.27)
**Motor Delay**		
Prenatal Cannabis Use		
No	2,992	1.00 (Ref)
Yes	137	0.81 (0.68 – 0.97)
Frequency of Prenatal Cannabis Use		
None	2,992	1.00 (Ref)
Monthly or Less	28	0.68 (0.47 – 0.99)
Weekly	15	0.76 (0.45 – 1.27)
Daily	12	0.81 (0.45 – 1.46)
Unknown Frequency[b]	82	0.87 (0.69 – 1.09)

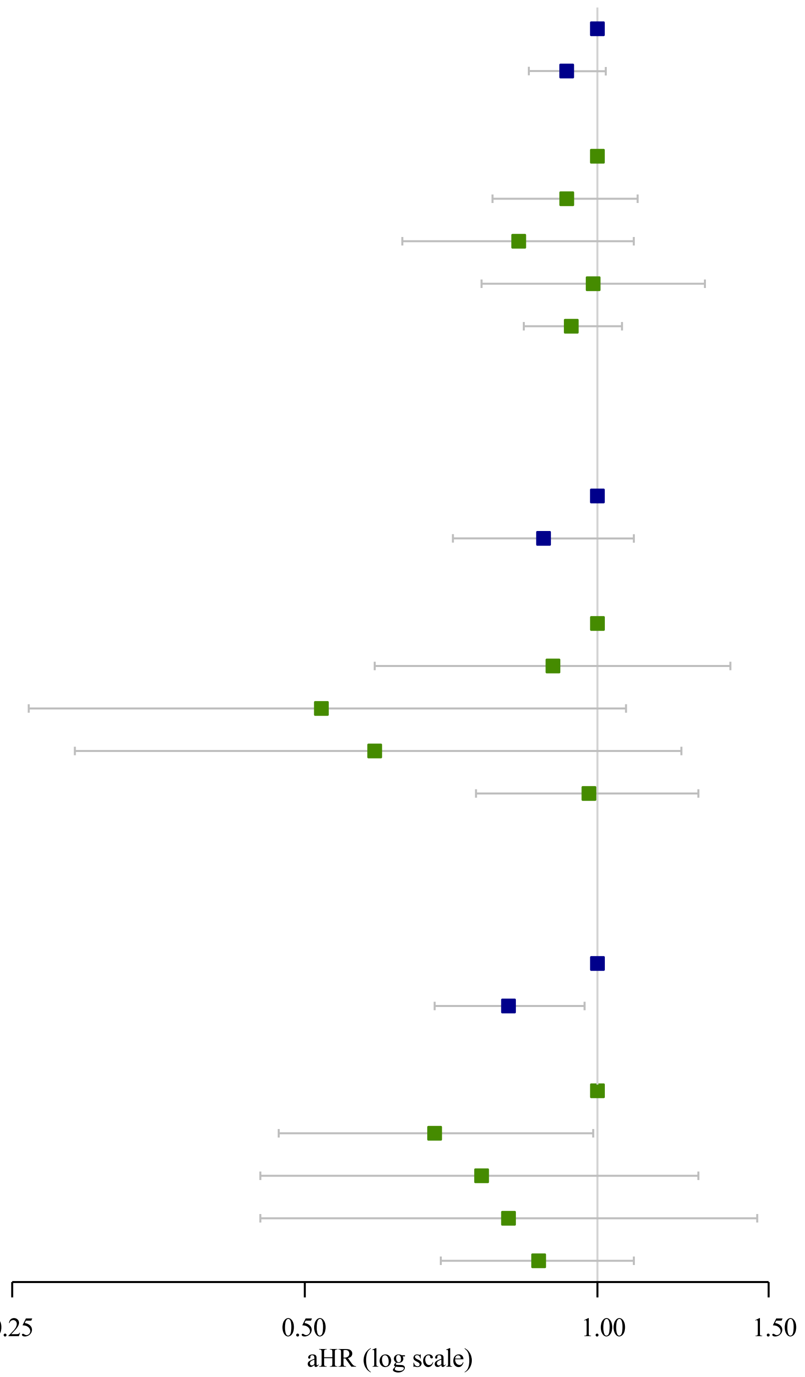

aHR= Adjusted Hazard Ratio; CI= Confidence Interval

[a]Adjusted for maternal sociodemographic characteristics, prenatal care initiation, and medical and mental health comorbidities [b]Positive urine toxicology test for Tetrahydrocannabinol (THC), but reported no cannabis use since becoming pregnant

© 2024 Avalos LA et al. *JAMA Network Open.*

eFigure 5. Sensitivity Analysis Cohort Assembly of Infants Born to Pregnant Individuals Screened for Prenatal Substance Use at Kaiser Permanente Northern California (KPNC) from 2015 to 2019, With No Continuous Enrollment Requirement and Either Prenatal Substance Use Screening Questionnaire or Prenatal Urine Toxicology Test Completed

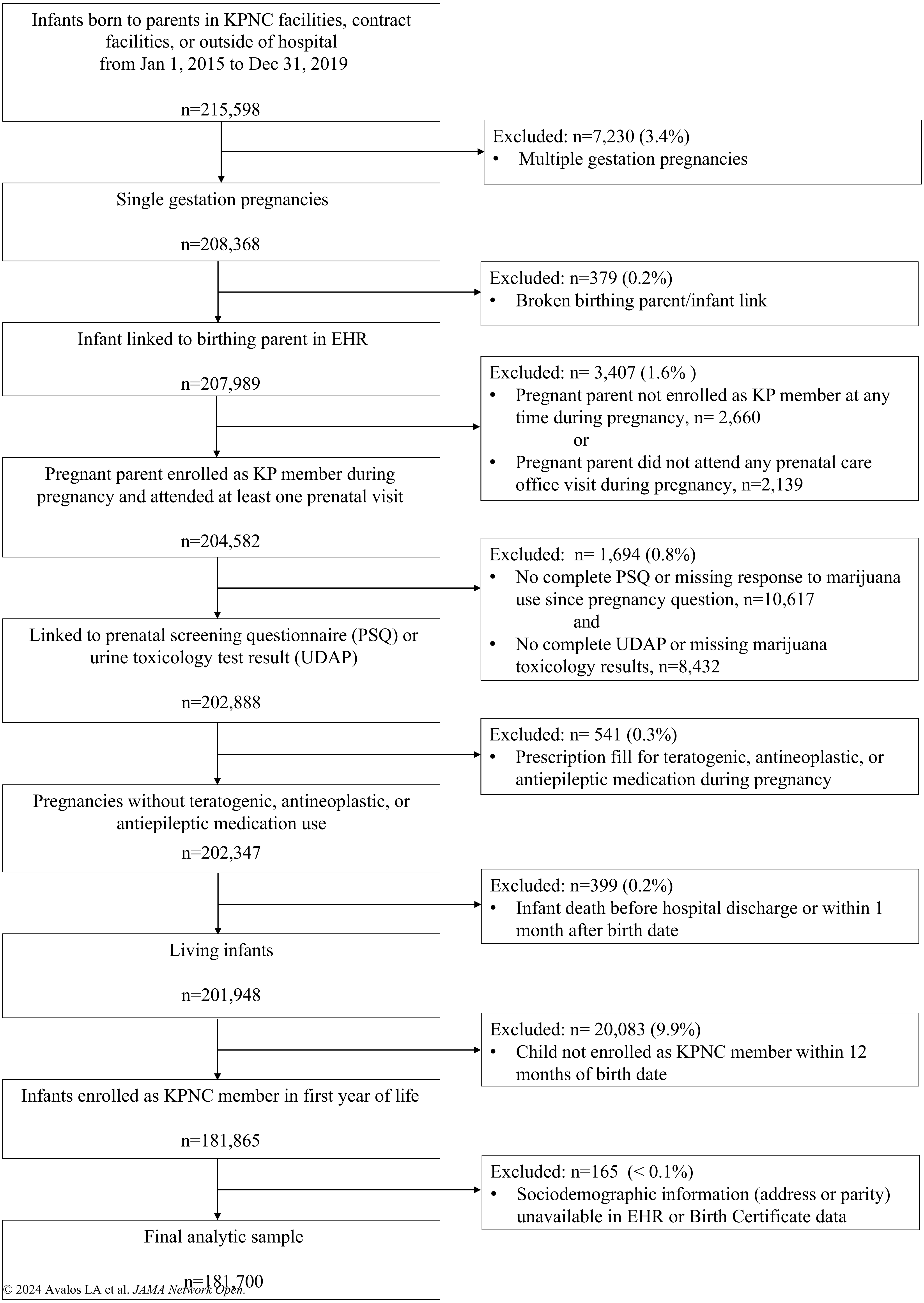

© 2024 Avalos LA et al. *JAMA Network Open.*
