# Supplementary material for: Early Maternal Prenatal Cannabis Use and Child Developmental Delays
Source: JAMA Netw Open. 2024 Oct 18;7(10):e2440295. doi: 10.1001/jamanetworkopen.2024.40295 (PMC11581621; doi:10.1001/jamanetworkopen.2024.40295)
Supplement: Supplement 2. — Data Sharing Statement [file jamanetwopen-e2440295-s002.pdf]

Data Sharing Statement

Avalos. Early Maternal Prenatal Cannabis Use and Child Developmental Delays. *JAMA Netw Open*. Published October 18, 2024. doi:10.1001/jamanetworkopen.2024.40295

Data
Data available: No
